# Deep phenotyping of heart failure with preserved ejection fraction through multi‐omics integration

**DOI:** 10.1002/ejhf.70041

**Published:** 2025-09-22

**Authors:** Jakob Versnjak, Titus Kuehne, Pauline Fahjen, Nina Jovanovic, Ulrike Löber, Gabriele G. Schiattarella, Nicola Wilck, Holger Gerhardt, Dominik N. Müller, Frank Edelmann, Philipp Mertins, Roland Eils, Michael Gotthardt, Sofia K. Forslund, Benjamin Wild, Marcus Kelm

**Affiliations:** ^1^ Institute of Computer‐assisted Cardiovascular Medicine, Deutsches Herzzentrum der Charité Berlin Germany; ^2^ Charité‐Universitätsmedizin Berlin, corporate member of Freie Universität Berlin and Humboldt‐Universität zu Berlin Berlin Germany; ^3^ DZHK (German Centre for Cardiovascular Research), partner site Berlin Berlin Germany; ^4^ Department of Congenital Heart Disease – Pediatric Cardiology Deutsches Herzzentrum der Charité Berlin Germany; ^5^ Proteomics Platform, Max‐Delbrück‐Center for Molecular Medicine Berlin Germany; ^6^ Max Delbrück Center for Molecular Medicine in the Helmholtz Association Berlin Germany; ^7^ Experimental and Clinical Research Center, a cooperation of Charité‐Universitätsmedizin Berlin and Max Delbrück Center for Molecular Medicine Berlin Germany; ^8^ Department of Cardiology, Angiology and Intensive Care Medicine Deutsches Herzzentrum der Charité (DHZC) Berlin Germany; ^9^ Translational Approaches in Heart Failure and Cardiometabolic Disease, Max Delbrück Center for Molecular Medicine in the Helmholtz Association (MDC) Berlin Germany; ^10^ Berlin Institute of Health at Charité – Universitätsmedizin Berlin Berlin Germany; ^11^ Health Data Science Unit, Heidelberg University Hospital and BioQuant Heidelberg Germany; ^12^ Translational Cardiology and Functional Genomics, Max Delbrück Center for Molecular Medicine in the Helmholtz Association Berlin Germany; ^13^ Institute of Cardiovascular Sciences, University College London London UK

**Keywords:** AI, artificial intelligence, Explainable artificial intelligence, Heart Failure Stage A: At Risk for Heart Failure, Heart Failure Stage B: Pre‐Heart Failure, Heart failure with preserved ejection fraction, Machine learning, Multi‐omics, Pre‐symptomatic heart failure

## Abstract

**Aims:**

Heart failure with preserved ejection fraction (HFpEF) has become the predominant form of heart failure and a leading cause of global cardiovascular morbidity and mortality. Due to its heterogeneous nature, HFpEF presents substantial challenges in diagnosis and management. Given the limited treatment options and lifestyle‐associated comorbidities, early identification is crucial for establishing effective preventive strategies. Here, we introduce and validate a machine learning‐based multi‐omics approach that integrates clinical and molecular data to detect and characterize HFpEF.

**Methods and results:**

A supervised classifier was trained on a stratified subset of UK Biobank participants (*n* = 401 917) to identify phenotypic profiles associated with subsequent symptom‐defined HFpEF during longitudinal follow‐up. Model performance was validated in a non‐overlapping hold‐out subset from all 22 UK Biobank assessment centres (*n* = 100 446; 6726 HFpEF cases; 7394 with multi‐omics data). The classifier demonstrated robust discriminatory performance, with a receiver operating characteristic area under the curve (ROC AUC) of 0.931 (95% confidence interval [CI] 0.930–0.931), a sensitivity of 0.857 (95% CI 0.855–0.860) and a specificity of 0.847 (95% CI 0.846–0.847). It identified individuals who subsequently developed HFpEF an average of 6.3 ± 3.9 years before symptom onset in asymptomatic individuals. Similarity network fusion (SNF) identified distinct subgroups, including a high‐risk cluster characterized by elevated mortality and dysregulated inflammatory pathways, which was distinguishable with high accuracy (ROC AUC 0.988; 95% CI 0.985–0.990).

**Conclusions:**

We identified HFpEF phenotypes at an early stage, often several years before the onset of clinical symptoms, when the disease trajectory may still be amenable to modification. The molecular characterization provides novel insights into the underlying disease complexity and enables more refined risk stratification.

## Introduction

Although heart failure with preserved ejection fraction (HFpEF) constitutes more than half of all heart failure cases, it remains underrecognized, posing challenges in diagnosis, risk stratification and therapeutic management.^1^, ^2^ HFpEF is a complex multifactorial syndrome, characterized by myocardial stiffness, impaired diastolic filling despite preserved left ventricular ejection fraction, along with vascular dysfunction and metabolic inflammation contributing to its heterogeneous clinical presentation and outcomes.^3^, ^4^ The condition is frequently associated with comorbidities such as obesity, hypertension and diabetes, further complicating the clinical picture and increasing the risk of an adverse disease course.^5^ Despite extensive research and evolving therapeutic approaches,^6^, ^7^, ^8^ effective treatments for HFpEF remain limited, highlighting the importance of early detection, preventive strategies, and individualized diagnostic and therapeutic strategies to address this unmet need in cardiovascular health.^7^, ^8^, ^9^

Recent advances in data‐driven methods offer promising solutions for HFpEF detection, including score‐based screening tools^10^, ^11^, ^12^, ^13^ and approaches incorporating molecular biomarkers.^14^, ^15^, ^16^ In this study, we aimed to utilize integrative multimodal data to enhance the early detection of HFpEF and improve risk stratification. Supervised machine learning classifiers were trained to identify individuals at risk of developing symptomatic HFpEF, and the models were validated in an independent multicentre cohort. Furthermore, unsupervised clustering techniques stratified HFpEF patients into distinct phenotype subgroups based on multi‐omics data. We applied explainable artificial intelligence (XAI) methods, combined with deconfounding techniques^17^ and pathway analysis, to elucidate the molecular heterogeneity of HFpEF.

## Methods

### Study participants

The UK Biobank is a cohort study comprising 502 366 individuals aged 40 to 69 years at enrolment. Biomedical data were collected, including proteomic and metabolomic profiles, routine clinical laboratory measurements, physical assessments, genetic information, questionnaires and environmental variables, with longitudinal follow‐up through electronic health records (EHRs). Baseline assessments were conducted at 22 centres across Scotland, England and Wales between 2006 and 2010.^18^, ^19^, ^20^ All information, including the UK Biobank Pharma Proteomics Project (UKB‐PPP), was accessed on 16 November 2023. All participants provided written informed consent, and the study was approved by the North West Multi‐Centre Research Ethics Committee (REC reference 11/NW/0382).

**Figure 1:**
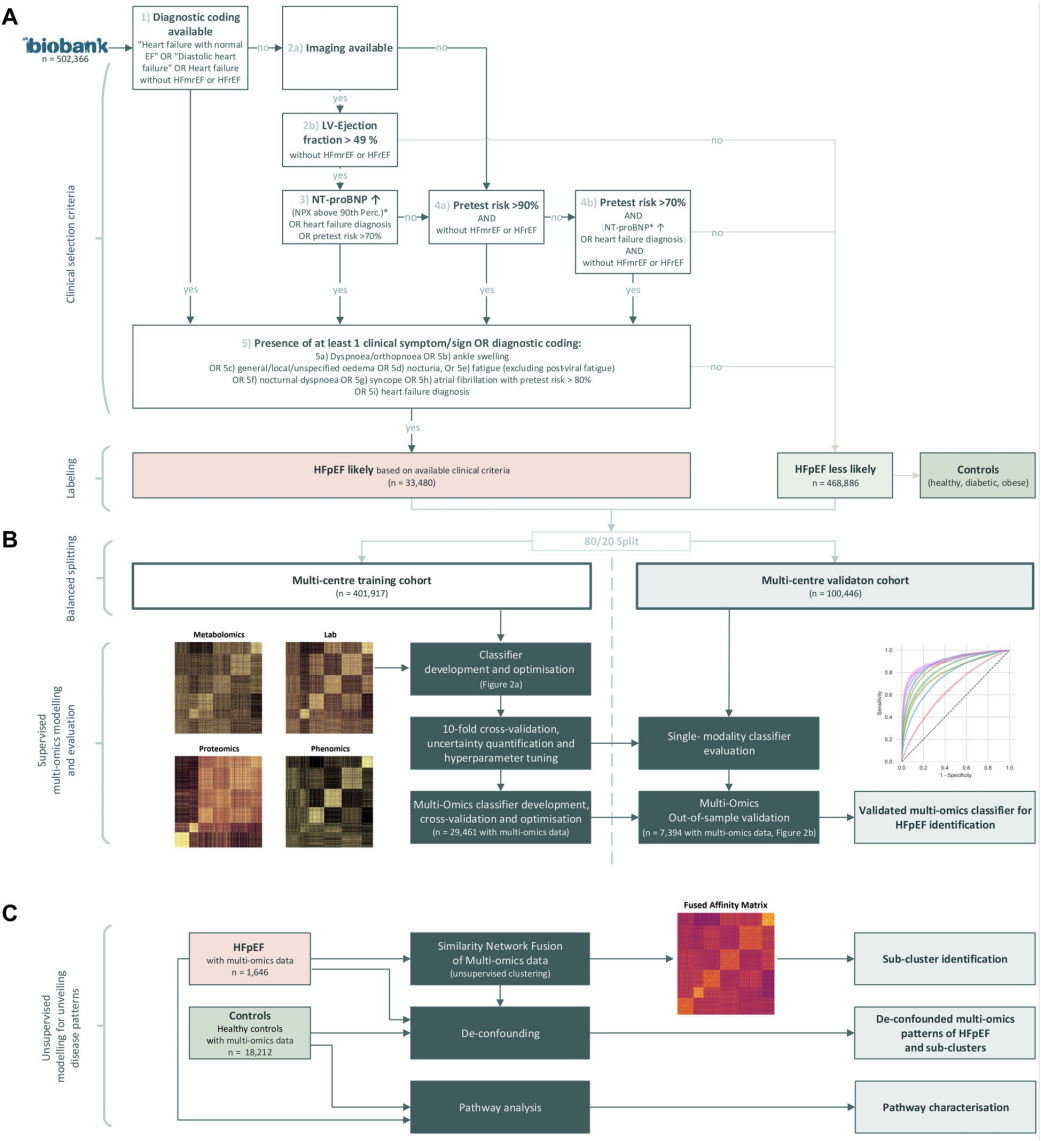
Patient selection and study design. (*A*) Flowchart illustrating the selection of heart failure with preserved ejection fraction (HFpEF) patients (*n* = 33 480) and control subjects based on clinical criteria. (*B*) Schematic representation of the supervised modeling pipeline, including a balanced 80/20 split of data into training and validation cohorts. (*C*) Overview of the unsupervised modeling approaches employed. EF, ejection fraction; HFmrEF, heart failure with mildly reduced ejection fraction; HFrEF, heart failure with reduced ejection fraction; LV, left ventricular; NPX, normalized protein expression. *If N‐terminal pro‐B‐type natriuretic peptide (NT‐proBNP) level was available.

The identification of patients with HFpEF followed a multi‐stage process, in which no single criterion alone was sufficient for classification (*Figure* *1* *A* and online supplementary *Table Appendix* *S1*). The primary inclusion criterion was the presence of at least one clinical symptom of heart failure as defined by the literature^3^, ^21^ or an associated diagnostic code (online supplementary *Methods*). When direct diagnostic codes were unavailable, further classification was either conceptually aligned with guideline‐based criteria,^3^ incorporating imaging data from cardiac magnetic resonance (CMR) imaging (available for 38 141–39 610 participants, depending on the parameter) and N‐terminal pro‐B‐type natriuretic peptide (NT‐proBNP) levels (available for 51 578 participants), or, for participants without available imaging data, an elevated pre‐test risk combined with available clinical criteria.^11^, ^22^, ^23^ Participants with a quality‐controlled left ventricular ejection fraction >49% and NT‐proBNP levels above the 90th percentile, defined using all participants with available measurements as a reference, were assigned to the HFpEF group, reflecting the elevated natriuretic peptide criteria recommended by the European Society of Cardiology (ESC) guidelines for heart failure.^3^ If imaging data were not available, the pre‐test risk was calculated using the validated H_2_FPEF score.^11^, ^22^ Subsequently, symptomatic participants with a very high pre‐test risk (above 90%) were directly assigned to the HFpEF group.^22^ Additionally, if the pre‐test risk exceeded 70%, participants who later became symptomatic were classified as HFpEF only if subsequent confirmatory criteria were also met, including an NT‐proBNP level above the 90th percentile and/or heart failure diagnostic coding, provided there was no conflicting evidence indicating heart failure with mildly reduced ejection fraction (HFmrEF) or reduced ejection fraction (HFrEF) from diagnostic codes or cardiac imaging data.^11^, ^22^, ^23^

Individuals who did not meet these criteria were considered less likely to have HFpEF. A control cohort was selected from this group, comprising healthy non‐heart failure (non‐HF), HFrEF, diabetic and obese controls. To minimize regional biases, both the training cohort and a pre‐specified multicentre validation cohort were derived from the UK Biobank using an 80/20 stratified split, with no overlap between subsets. The study adhered to the Transparent Reporting of a Multivariable Prediction Model for Individual Prognosis or Diagnosis (TRIPOD) statement.^24^

### Data modalities

Phenomics data included 13 physiological measurements, such as body mass index (BMI), weight, waist circumference, blood pressure, and pulse rate. Sex and age were excluded from the unsupervised clustering algorithm to determine if the resulting subgroups would exhibit distinct distributions of these characteristics. However, these two variables were included in subsequent risk factor identification to assess their effects.

Metabolomics data were derived for 483 980 UK Biobank participants, including 251 metabolite biomarkers profiled from plasma samples using a nuclear magnetic resonance (NMR)‐based metabolomic biomarker profiling platform. The dataset encompassed lipoprotein lipids, fatty acids, amino acids, ketone bodies and metabolites involved in glycolysis.

Laboratory data comprised assays from blood samples, totaling 55 features. Proteomic profiling was conducted on a randomized subset of 54 306 UK Biobank participants using Olink Proximity Extension assays. The dataset included the expressions of 2923 proteins across cardiometabolic, inflammation, neurology and oncology panels.^18^ Protein expressions were provided in the normalized protein expression (NPX) format.

Genetic information included aggregated polygenic risk scores (PRSs), leucocyte telomere length and single‐nucleotide polymorphisms (SNPs). Thirty‐six standard PRSs for disease and quantitative traits were included, derived from external genome‐wide association study (GWAS) data.^25^ Relative leucocyte telomere length, adjusted for technical parameters, was also included.^26^ From the phenotype–genotype reference map,^27^ 28 out of 5879 SNPs were selected for their predictive power in classifying HFpEF versus non‐HF controls (online supplementary *Methods*).

Variables related to medication intake, disease diagnoses and symptoms were also included. These variables were identified using ICD‐10 encodings, phecodes^28^ (online supplementary *Table* *S2*) and encodings based on the Observational Medical Outcomes Partnership (OMOP) Common Data Model (CDM) (online supplementary *Tables* *S3* and S1). Only EHR data logged prior to recruitment were considered as model input. Additionally, responses to touchscreen questionnaires at recruitment, such as sociodemographic, lifestyle, environment and psychosocial factors, were added. Multiple questionnaire items were condensed into single variables to reduce the number of features.

### Machine learning approaches

The unsupervised similarity network fusion (SNF) algorithm was employed to cluster multi‐omics data from the HFpEF cohort, combining metabolomics, clinical laboratory measurements, proteomics and phenomics (Figure *1C*). SNF demonstrated superior performance compared with concatenated or single‐modality analyses^29^ (online supplementary *Methods*). Associated code and scripts for the SNF analysis can be found in the following GitHub repository: https://github.com/charite‐icm/snf.

Supervised CatBoost (v1.2.3)^30^ was employed in Python as the case‐control classifier, using nested two‐level 10‐fold cross‐validation (Figure *1B*). Each fold was stratified to preserve the same percentage of disease cases across all folds. CatBoost's ability to handle missing data allowed the inclusion of samples with incomplete features. Shapley Additive exPlanations (SHAP) values were calculated using the Python package shap (v0.44.0). SHAP quantifies the contribution of each feature to individual predictions. SHAP values were also calculated for the CatBoost classifiers trained to predict clusters identified by SNF against control groups, which included HFpEF patients not assigned to the cluster and non‐HFpEF individuals (online supplementary *Methods*).

### Statistical analyses and control of confounding

To evaluate the discriminatory performance, accuracy and calibration of the supervised machine learning models, receiver operating characteristic areas under the curve (ROC AUCs), classification accuracy and Brier scores were employed. No imputation was performed for missing variables. Continuous data are expressed as mean ± standard deviation (SD) unless stated otherwise, while categorical data are presented as frequencies and percentages. Unadjusted *p*‐values are reported in the baseline tables for descriptive purposes only, without correction for multiple testing. Data distribution was evaluated using the Shapiro–Wilk and Shapiro–Francia tests. Differences in continuous variables between groups were analysed using either the main effects test from a linear regression or the Mann–Whitney U test. Categorical variables were compared using Pearson's *χ* ^2^ test or Fisher's exact test, as appropriate. ROC AUCs were compared against references (HFpEF‐ABA score and NT‐proBNP) using DeLong's method. All statistical analyses were conducted using Python (v3.11) and Stata (v18.0 MP).

To identify omics features associated with control and disease groups without being affected by confounding factors, the R package metadeconfoundR (v0.3.0) was employed. Initially, non‐parametric tests were applied to detect naïve associations between omics features and covariates (e.g. medications, diseases) in cross‐sectional datasets using the Mann–Whitney U test for binary features and Spearman's test for continuous features. Results were adjusted for multiple testing using the Benjamini–Hochberg method, and standardized effect sizes (Cliff's delta, Spearman's ρ) were calculated. Significant omics features underwent post‐hoc nested linear model comparisons, with likelihood ratio tests (LRT) assessing covariate effects. Features were considered deconfounded if disease status provided additional predictive power beyond each covariate (LRT <0.05); otherwise, they were classified as confounded.^17^, ^31^ The analysis included 3241 omics features derived from laboratory data, phenomics (including age and sex), metabolomics and proteomics data. These features were tested against 134 potential confounders, such as medication and vitamin intake, diagnoses, symptoms and responses from online questionnaires.

### Gene set enrichment analysis

Single‐sample gene set enrichment analysis (ssGSEA, ssGSEA2.0) was used to assess changes in pathway activity. The input for ssGSEA consisted of effect sizes derived from the confounding analysis for HFpEF and its subclusters compared to non‐HF controls. Gene sets were sourced from gene ontology biological pathways (c5.go.bp.v2023.2.Hs.symbols.gmt) and curated pathways (c2.cgp.v2023.2.Hs.symbols.gmt) from the molecular signature database (MSigDB). A pathway was considered cluster‐specific if it met a false discovery rate (FDR) cut‐off of 0.05, had an enrichment score of at least 1 SD from the pathway mean and exhibited the maximum or minimum value for that cluster. Redundant pathways showing more than 50% overlap of enriched genes with a higher‐scoring pathway were removed.

## Results

### Study population

Multi‐stage selection (*Figure* *1A*) resulted in 33 480 HFpEF cases and 468 886 non‐HFpEF individuals, including 256 895 non‐HF controls, 10 247 diabetic controls and 38 772 obese controls. Additionally, 486 individuals with HFrEF and available imaging data were identified (*Table* *1* and online supplementary *Table* *S5*). Median observational follow‐up time was 13.95 years (interquartile range 13.20–14.67), with a maximum of 16.94 years. Total person‐time amounted to 6 829 239 person‐years, with 44 499 deaths recorded across the full cohort. For the age group studied, the estimated HFpEF prevalence closely matched earlier reported findings.^1^ The pre‐specified multicentre cohort served as a holdout set for out‐of‐sample evaluation. The dataset was split 80/20 into a training (*n* = 401 917) and an independent validation cohort (*n* = 100 446), stratified to ensure balanced distributions of key clinical characteristics. Three cases lacking clearly defined stratification criteria were excluded from the analysis. This yielded 26 754 HFpEF patients in the training and 6726 patients in the validation cohort. Distributions of diabetes, obesity, sex, age, HFrEF and HFpEF were comparable across cohorts (*Table* *2*).

**Table 1:** Baseline characteristics of the heart failure with preserved ejection fraction (HFpEF) cohort and control groups

	Non‐HF controls (*n* = 256 895)	Obese controls (*n* = 38 772)	Diabetic controls (*n* = 10 247)	HFpEF (*n* = 33 480)	*p*‐value
Age at recruitment (years)	54.949 (8.022)	50.861 (5.893)	57.802 (7.468)	63.278 (5.166)	<0.001
Female sex	147 518 (57.4)	21 570 (55.6)	4155 (40.5)	13 161 (39.3)	<0.001
Female before menopause	48 989 (19.1)	9768 (25.2)	823 (8.0)	652 (1.9)	<0.001
Waist circumference (cm)	84.469 (10.207)	102.611 (10.216)	91.394 (9.511)	101.778 (14.133)	<0.001
Body mass index (kg/m^2^)	25.110 (2.708)	33.509 (3.529)	26.532 (2.426)	31.240 (5.539)	<0.001
Pulse rate (bpm)	68.256 (10.507)	71.985 (10.893)	72.196 (11.762)	69.661 (13.173)	<0.001
Mortality (all‐cause)	11 548 (4.5)	1305 (3.4)	1166 (11.4)	9371 (28.0)	<0.001
Systolic blood pressure (mmHg)	134.951 (18.157)	137.589 (16.366)	140.626 (18.102)	144.650 (19.420)	<0.001
Diastolic blood pressure (mmHg)	80.896 (9.867)	86.387 (9.493)	82.236 (9.939)	83.098 (10.712)	<0.001
Pulse pressure (mmHg)	54.055 (12.745)	51.202 (11.342)	58.390 (13.769)	61.550 (15.580)	<0.001
Sleep apnoea	487 (0.2)	224 (0.6)	75 (0.7)	568 (1.7)	<0.001
Chronic ischaemic heart disease	1883 (0.7)	189 (0.5)	384 (3.7)	5074 (15.2)	<0.001
Non‐rheumatic mitral valve disease	139 (0.1)	7 (0.0)	8 (0.1)	547 (1.6)	<0.001
Non‐rheumatic aortic valve disease	108 (0.0)	15 (0.0)	10 (0.1)	489 (1.5)	<0.001
Cardiomyopathy	28 (0.0)	9 (0.0)	2 (0.0)	355 (1.1)	<0.001
Varicose veins of lower extremities	4618 (1.8)	666 (1.7)	165 (1.6)	840 (2.5)	<0.001
Hypotension	343 (0.1)	41 (0.1)	33 (0.3)	353 (1.1)	<0.001
Angina pectoris/coronary artery disease	1561 (0.6)	185 (0.5)	288 (2.8)	3843 (11.5)	<0.001
Endocrine, nutritional and metabolic diseases	6579 (2.6)	1590 (4.1)	2412 (23.5)	8116 (24.2)	<0.001
Mental and behavioural disorders	3516 (1.4)	810 (2.1)	362 (3.5)	1461 (4.4)	<0.001
Diseases of the nervous system	7745 (3.0)	1767 (4.6)	644 (6.3)	3322 (9.9)	<0.001
Diseases of the eye and adnexa	8308 (3.2)	990 (2.6)	761 (7.4)	2848 (8.5)	<0.001
Diseases of the respiratory system	12 292 (4.8)	2486 (6.4)	866 (8.5)	5221 (15.6)	<0.001
Diseases of the digestive system	43 157 (16.8)	7279 (18.8)	2779 (27.1)	11 466 (34.2)	<0.001
Diseases of the skin and subcutaneous tissue	11 278 (4.4)	2041 (5.3)	680 (6.6)	3179 (9.5)	<0.001
Diseases of the musculoskeletal system and connective tissue	26 477 (10.3)	4865 (12.5)	1720 (16.8)	8800 (26.3)	<0.001
Diseases of the genitourinary system	35 332 (13.8)	6051 (15.6)	1686 (16.5)	6842 (20.4)	<0.001
Pregnancy, childbirth and the puerperium	12 029 (4.7)	1974 (5.1)	158 (1.5)	64 (0.2)	<0.001
Aortic aneurysm	60 (0.0)	3 (0.0)	6 (0.1)	189 (0.6)	<0.001
Phlebitis and thrombophlebitis	1268 (0.5)	271 (0.7)	80 (0.8)	948 (2.8)	<0.001
Type 2 diabetes	0 (0.0)	0 (0.0)	2628 (25.6)	3820 (11.4)	<0.001
Myocardial infarction	883 (0.3)	72 (0.2)	158 (1.5)	2555 (7.6)	<0.001
Chronic kidney disease	940 (0.4)	103 (0.3)	117 (1.1)	2055 (6.1)	<0.001
Hypertensive renal disease	73 (0.0)	7 (0.0)	21 (0.2)	305 (0.9)	<0.001
Endocarditis	370 (0.1)	42 (0.1)	14 (0.1)	294 (0.9)	<0.001
Essential (primary) hypertension	6349 (2.5)	1102 (2.8)	1216 (11.9)	10 217 (30.5)	<0.001
Atrial fibrillation and flutter	0 (0.0)	0 (0.0)	0 (0.0)	5962 (17.8)	<0.001
Cerebrovascular event/stroke	1053 (0.4)	149 (0.4)	127 (1.2)	1371 (4.1)	<0.001
Embolism and thrombosis	2436 (0.9)	448 (1.2)	154 (1.5)	1691 (5.0)	<0.001
Pulmonary embolism	504 (0.2)	111 (0.3)	30 (0.3)	466 (1.4)	<0.001
Pulmonary arterial hypertension	22 (0.0)	4 (0.0)	2 (0.0)	123 (0.4)	<0.001
Neoplasms	33 946 (13.2)	4750 (12.3)	1591 (15.5)	6640 (19.8)	<0.001
Congenital malformations of the circulatory system	221 (0.1)	31 (0.1)	9 (0.1)	189 (0.6)	<0.001
Seizure/epilepsy	1565 (0.6)	276 (0.7)	107 (1.0)	556 (1.7)	<0.001
Swollen ankle region	0 (0.0)	0 (0.0)	0 (0.0)	1212 (3.6)	<0.001
Impaired exercise tolerance	0 (0.0)	0 (0.0)	0 (0.0)	1448 (4.3)	<0.001
Joint swelling	2 (0.0)	0 (0.0)	0 (0.0)	4 (0.0)	<0.001
Tachycardia	146 (0.1)	28 (0.1)	18 (0.2)	134 (0.4)	<0.001
Bradycardia	183 (0.1)	23 (0.1)	15 (0.1)	331 (1.0)	<0.001
Palpitations	515 (0.2)	92 (0.2)	36 (0.4)	585 (1.7)	<0.001
Cough	351 (0.1)	73 (0.2)	24 (0.2)	241 (0.7)	<0.001
Symptoms and signs involving the digestive system and abdomen	14 892 (5.8)	2587 (6.7)	895 (8.7)	3695 (11.0)	<0.001
Symptoms and signs involving the skin and subcutaneous tissue	1418 (0.6)	292 (0.8)	107 (1.0)	516 (1.5)	<0.001
Symptoms and signs involving the urinary system	6912 (2.7)	929 (2.4)	468 (4.6)	2414 (7.2)	<0.001
Symptoms and signs involving cognition, perception, emotional state and behaviour	964 (0.4)	184 (0.5)	85 (0.8)	631 (1.9)	<0.001
Symptoms and signs involving speech and voice	406 (0.2)	70 (0.2)	35 (0.3)	233 (0.7)	<0.001
Dyspnoea	0 (0.0)	0 (0.0)	0 (0.0)	3537 (10.6)	<0.001
General, local, unspecified oedema	0 (0.0)	0 (0.0)	0 (0.0)	163 (0.5)	<0.001
Nocturia	0 (0.0)	0 (0.0)	0 (0.0)	459 (1.4)	<0.001
Post‐viral fatigue	794 (0.3)	84 (0.2)	25 (0.2)	130 (0.4)	<0.001
Syncope	0 (0.0)	0 (0.0)	0 (0.0)	1574 (4.7)	<0.001
Fatigue (excl. post‐viral)	18 (0.0)	5 (0.0)	2 (0.0)	2510 (7.5)	<0.001
Cholesterol‐lowering medication	20 230 (7.9)	2865 (7.4)	4428 (42.5)	15 511 (46.3)	<0.001
Mineralocorticoid receptor antagonist	52 (0.0)	6 (0.0)	4 (0.0)	367 (1.1)	<0.001
Levothyroxine	2732 (1.1)	452 (1.2)	151 (1.5)	1364 (4.1)	<0.001
Metformin	64 (0.0)	29 (0.1)	575 (5.6)	1696 (5.1)	<0.001
Warfarin	308 (0.1)	66 (0.2)	12 (0.1)	1916 (5.7)	<0.001
Sulfonylurea	27 (0.0)	4 (0.0)	324 (3.2)	931 (2.8)	<0.001
Iron therapy	4985 (1.9)	659 (1.7)	185 (1.8)	1448 (4.3)	<0.001
Beta‐blocker	10 441 (4.1)	1111 (2.9)	832 (8.1)	12 954 (38.7)	<0.001
ACE inhibitor	11 607 (4.5)	1688 (4.4)	2005 (19.6)	13 237 (39.5)	<0.001
Angiotensin receptor blocker	3753 (1.5)	504 (1.3)	612 (6.0)	5292 (15.8)	<0.001
Loop diuretic	0 (0.0)	0 (0.0)	0 (0.0)	3846 (11.5)	<0.001
Calcium channel blocker	8825 (3.4)	876 (2.3)	926 (9.0)	10 150 (30.3)	<0.001
Aspirin	19 790 (7.7)	2781 (7.2)	2841 (27.7)	14 238 (42.5)	<0.001
Thiazide diuretic	6479 (2.5)	533 (1.4)	573 (5.6)	8988 (26.8)	<0.001

Continuous variables are presented as mean (standard deviation), while binary variables are shown as *n* (%). Between‐group differences in continuous variables were analysed using either linear regression main effects testing or the Kruskal–Wallis rank test. Categorical variables were compared using Pearson's *χ* ^2^ test or Fisher's exact test, as appropriate.

ACE, angiotensin‐converting enzyme; HF, heart failure; HFpEF, heart failure with preserved ejection fraction.

**Table 2:** Baseline characteristics of the training and validation cohorts

	Multicentre training cohort (*n* = 401 917)	Multicentre validation cohort (*n* = 100 446)	Total (*n* = 502 363)	*p*‐value
Assessment centre				
Stockport (pilot)	3016 (0.8)	775 (0.8)	3791 (0.8)	0.481
Manchester	11 138 (2.8)	2799 (2.8)	13 937 (2.8)	
Oxford	11 225 (2.8)	2829 (2.8)	14 054 (2.8)	
Cardiff	14 293 (3.6)	3582 (3.6)	17 875 (3.6)	
Glasgow	14 985 (3.7)	3659 (3.6)	18 644 (3.7)	
Edinburgh	13 828 (3.4)	3365 (3.4)	17 193 (3.4)	
Stoke	15 536 (3.9)	3890 (3.9)	19 426 (3.9)	
Reading	23 627 (5.9)	5772 (5.7)	29 399 (5.9)	
Bury	22 629 (5.6)	5681 (5.7)	28 310 (5.6)	
Newcastle	29 712 (7.4)	7283 (7.3)	36 995 (7.4)	
Leeds	35 196 (8.8)	8988 (8.9)	44 184 (8.8)	
Bristol	34 337 (8.5)	8663 (8.6)	43 000 (8.6)	
Barts	10 088 (2.5)	2485 (2.5)	12 573 (2.5)	
Nottingham	27 113 (6.7)	6757 (6.7)	33 870 (6.7)	
Sheffield	24 181 (6.0)	6200 (6.2)	30 381 (6.0)	
Liverpool	26 188 (6.5)	6610 (6.6)	32 798 (6.5)	
Middlesborough	17 029 (4.2)	4254 (4.2)	21 283 (4.2)	
Hounslow	23 153 (5.8)	5713 (5.7)	28 866 (5.7)	
Croydon	21 972 (5.5)	5391 (5.4)	27 363 (5.4)	
Birmingham	20 333 (5.1)	5159 (5.1)	25 492 (5.1)	
Swansea	1825 (0.5)	455 (0.5)	2280 (0.5)	
Wrexham	513 (0.1)	136 (0.1)	649 (0.1)	
Age at recruitment (years)	56.530 (8.095)	56.531 (8.094)	56.530 (8.095)	0.958
Female sex	218 651 (54.4)	54 646 (54.4)	273 297 (54.4)	0.994
Female before menopause	61 477 (15.3)	15 287 (15.2)	76 764 (15.3)	0.545
Waist circumference (cm)	90.323 (13.493)	90.267 (13.458)	90.312 (13.486)	0.239
Standing height (cm)	168.445 (9.277)	168.440 (9.287)	168.444 (9.279)	0.882
Seated height (cm)	136.967 (7.174)	136.934 (7.195)	136.960 (7.178)	0.204
Body mass index (kg/m^2^)	27.436 (4.804)	27.421 (4.799)	27.433 (4.803)	0.386
Mortality (all‐cause)	35 779 (8.9)	8720 (8.7)	44 499 (8.9)	0.028
Pulse rate (bpm)	69.428 (11.260)	69.401 (11.278)	69.423 (11.264)	0.486
Systolic blood pressure (mmHg)	137.862 (18.659)	137.904 (18.631)	137.870 (18.653)	0.517
Diastolic blood pressure (mmHg)	82.261 (10.150)	82.239 (10.133)	82.257 (10.147)	0.536
Non‐HF controls	205 402 (51.1)	51 493 (51.3)	256 895 (51.1)	0.368
Diabetic controls	8224 (2.0)	2023 (2.0)	10 247 (2.0)	0.519
Obese controls	31 116 (7.7)	7656 (7.6)	38 772 (7.7)	0.203
HFpEF	26 754 (6.7)	6726 (6.7)	33 480 (6.7)	0.655
Sleep apnoea	1968 (0.5)	511 (0.5)	2479 (0.5)	0.440
Chronic ischaemic heart disease	12 631 (3.1)	3181 (3.2)	15 812 (3.1)	0.695
Non‐rheumatic mitral valve disease	853 (0.2)	212 (0.2)	1065 (0.2)	0.942
Non‐rheumatic aortic valve disease	853 (0.2)	195 (0.2)	1048 (0.2)	0.261
Cardiomyopathy	502 (0.1)	115 (0.1)	617 (0.1)	0.399
Varicose veins of lower extremities	7784 (1.9)	2009 (2.0)	9793 (1.9)	0.194
Hypotension	1170 (0.3)	317 (0.3)	1487 (0.3)	0.201
Angina pectoris/coronary artery disease	9827 (2.4)	2475 (2.5)	12 302 (2.4)	0.728
Endocrine, nutritional and metabolic diseases	30 250 (7.5)	7517 (7.5)	37 767 (7.5)	0.645
Mental and behavioural disorders	9253 (2.3)	2336 (2.3)	11 589 (2.3)	0.658
Diseases of the nervous system	19 856 (4.9)	4899 (4.9)	24 755 (4.9)	0.409
Diseases of the eye and adnexa	17 621 (4.4)	4355 (4.3)	21 976 (4.4)	0.501
Diseases of the respiratory system	29 796 (7.4)	7396 (7.4)	37 192 (7.4)	0.586
Diseases of the digestive system	87 128 (21.7)	21 848 (21.8)	108 976 (21.7)	0.616
Diseases of the skin and subcutaneous tissue	22 757 (5.7)	5618 (5.6)	28 375 (5.6)	0.396
Diseases of the musculoskeletal system and connective tissue	57 836 (14.4)	14 404 (14.3)	72 240 (14.4)	0.686
Diseases of the genitourinary system	63 864 (15.9)	16 038 (16.0)	79 902 (15.9)	0.551
Pregnancy, childbirth and the puerperium	14 102 (3.5)	3592 (3.6)	17 694 (3.5)	0.300
Congenital malformations, deformations and chromosomal abnormalities	2899 (0.7)	744 (0.7)	3643 (0.7)	0.517
Phlebitis and thrombophlebitis	4227 (1.1)	1075 (1.1)	5302 (1.1)	0.607
Type 2 diabetes	12 215 (3.0)	3080 (3.1)	15 295 (3.0)	0.654
Myocardial infarction	6251 (1.6)	1561 (1.6)	7812 (1.6)	0.978
Chronic kidney disease	5932 (1.5)	1545 (1.5)	7477 (1.5)	0.145
Hypertensive renal disease	635 (0.2)	160 (0.2)	795 (0.2)	0.926
Endocarditis	1106 (0.3)	286 (0.3)	1392 (0.3)	0.607
Essential (primary) hypertension	32 349 (8.0)	8053 (8.0)	40 402 (8.0)	0.743
Atrial fibrillation and flutter	7062 (1.8)	1779 (1.8)	8841 (1.8)	0.762
Cerebrovascular event/stroke	4362 (1.1)	1079 (1.1)	5441 (1.1)	0.761
Embolism and thrombosis	7788 (1.9)	1911 (1.9)	9699 (1.9)	0.468
Pulmonary embolism	1808 (0.4)	407 (0.4)	2215 (0.4)	0.056
Pulmonary arterial hypertension	186 (0.0)	49 (0.0)	235 (0.0)	0.743
Neoplasms	60 109 (15.0)	15 115 (15.0)	75 224 (15.0)	0.463
Congenital malformations of the circulatory system	608 (0.2)	153 (0.2)	761 (0.2)	0.939
Seizure/epilepsy	3934 (1.0)	989 (1.0)	4923 (1.0)	0.867
Swollen ankle region	3451 (0.9)	828 (0.8)	4279 (0.9)	0.290
Impaired exercise tolerance	7167 (1.8)	1850 (1.8)	9017 (1.8)	0.211
Joint swelling	8 (0.0)	2 (0.0)	10 (0.0)	1.000
Tachycardia	493 (0.1)	130 (0.1)	623 (0.1)	0.586
Bradycardia	876 (0.2)	222 (0.2)	1098 (0.2)	0.853
Palpitations	1851 (0.5)	427 (0.4)	2278 (0.5)	0.135
Cough	1107 (0.3)	287 (0.3)	1394 (0.3)	0.579
Symptoms and signs involving the digestive system and abdomen	30 731 (7.6)	7567 (7.5)	38 298 (7.6)	0.229
Symptoms and signs involving the skin and subcutaneous tissue	3381 (0.8)	845 (0.8)	4226 (0.8)	0.999
Symptoms and signs involving the urinary system	14 855 (3.7)	3759 (3.7)	18 614 (3.7)	0.487
Symptoms and signs involving cognition, perception, emotional state and behaviour	3068 (0.8)	745 (0.7)	3813 (0.8)	0.479
Symptoms and signs involving speech and voice	1167 (0.3)	264 (0.3)	1431 (0.3)	0.143
Dyspnoea	12 379 (3.1)	3051 (3.0)	15 430 (3.1)	0.485
General, local, unspecified oedema	389 (0.1)	98 (0.1)	487 (0.1)	0.943
Nocturia	1791 (0.4)	454 (0.5)	2245 (0.4)	0.787
Post‐viral fatigue	1516 (0.4)	352 (0.4)	1868 (0.4)	0.213
Syncope	7625 (1.9)	1893 (1.9)	9518 (1.9)	0.794
Fatigue (excl. post‐viral)	16 714 (4.2)	4246 (4.2)	20 960 (4.2)	0.331
Cholesterol‐lowering medication	69 424 (17.3)	17 434 (17.4)	86 858 (17.3)	0.529
Mineralocorticoid receptor antagonist	680 (0.2)	171 (0.2)	851 (0.2)	0.942
Levothyroxine	8960 (2.2)	2243 (2.2)	11 203 (2.2)	0.943
Metformin	4853 (1.2)	1216 (1.2)	6069 (1.2)	0.935
Warfarin	2993 (0.7)	744 (0.7)	3737 (0.7)	0.895
Sulfonylurea	2551 (0.6)	618 (0.6)	3169 (0.6)	0.486
Iron therapy	12 385 (3.1)	3178 (3.2)	15 563 (3.1)	0.178
Beta‐blocker	44 837 (11.2)	11 334 (11.3)	56 171 (11.2)	0.250
ACE inhibitor	50 973 (12.7)	12 713 (12.7)	63 686 (12.7)	0.825
Angiotensin receptor blocker	18 557 (4.6)	4769 (4.7)	23 326 (4.6)	0.078
Loop diuretic	7801 (1.9)	1882 (1.9)	9683 (1.9)	0.165
Calcium channel blocker	37 802 (9.4)	9529 (9.5)	47 331 (9.4)	0.430
Aspirin	62 062 (15.4)	15 595 (15.5)	77 657 (15.5)	0.509
Thiazide diuretic	34 837 (8.7)	8674 (8.6)	43 511 (8.7)	0.745

Continuous variables are presented as mean (standard deviation), while binary variables are shown as *n* (%). Between‐group differences in continuous variables were analysed using either linear regression main effects testing or the Kruskal–Wallis rank test. Categorical variables were compared using Pearson's *χ* ^2^ test or Fisher's exact test, as appropriate.

ACE, angiotensin‐converting enzyme; HF, heart failure; HFpEF, heart failure with preserved ejection fraction.

### Model evaluation

**Figure 2:**
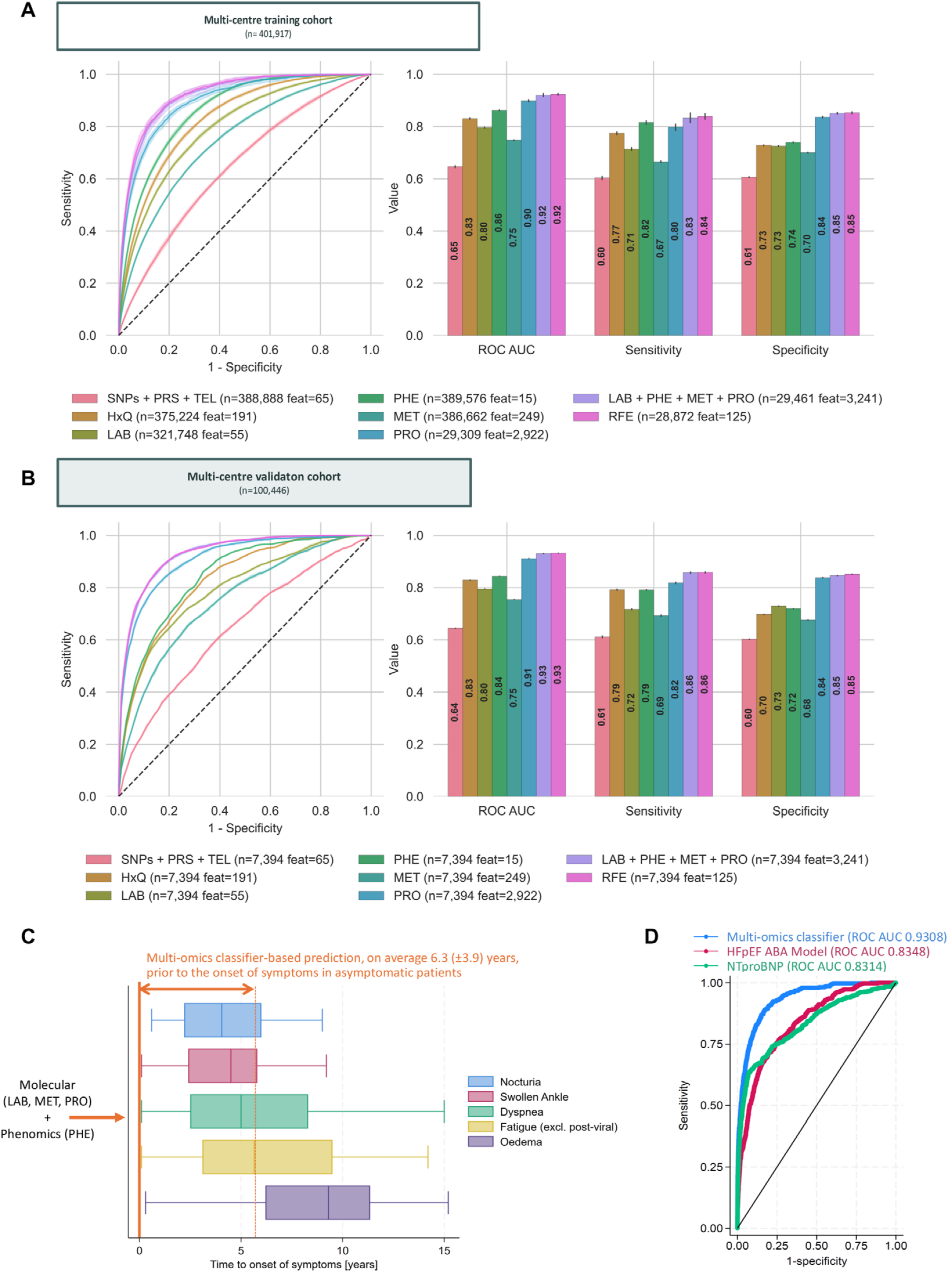
Performance metrics for machine learning classification. (*A*) Evaluation of the CatBoost classifiers using nested two‐level 10‐fold cross‐validation on the training cohort. The left subfigure depicts the mean receiver operating characteristic (ROC) curve, with the shaded area representing the 95% confidence interval. The bar plots on the right show the mean values for the ROC area under the curve (AUC), sensitivity and specificity, with error bars indicating the 95% confidence interval. Each cross‐validation experiment included varying numbers of cases (*n*) and features (feat) for different modality combinations, encoded by colour. (*B*) Performance evaluation of the classifiers on the validation cohort. The same validation cohort (*n* = 7394) was used for all modalities. (*C*) Boxplot illustrating the time of onset of heart failure symptoms in asymptomatic patients. (*D*) Comparison of the ROC AUC for three models tested on the validation cohort: (1) multi‐omics CatBoost classifier (LAB + PHE + MET + PRO); (2) HFpEF‐ABA reference model using only age, body mass index and atrial fibrillation as features; and (3) a simple model only using N‐terminal pro‐B‐type natriuretic peptide (NT‐proBNP) level with various thresholds to predict HFpEF.

Supervised machine learning classifiers were trained and optimized through hyperparameter tuning on a cohort of 26 754 HFpEF cases and 375 163 non‐HFpEF controls. Ten‐fold nested cross‐validation was performed to assess classifier performance, with metrics including ROC AUC, sensitivity and specificity (*Figure* *2A*). Classifiers included in the evaluation incorporated genomic information, questionnaires, medical history, laboratory measurements, phenomics, metabolomics and proteomics, all collected at or before the recruitment assessment. Data availability varied across modalities, resulting in differing sample sizes for each modality configuration. Participants without NT‐proBNP measurements were not included in multi‐omics analyses but were used for single modality analyses where NT‐proBNP was not required.

The best‐performing model configuration included laboratory data, phenomics, metabolomics and proteomics (LAB + PHE + MET + PRO), encompassing 29 461 training cases and 3241 omics features (*Figure* *2A* and online supplementary *Table* *S6*), yielding a ROC AUC of 0.921 (95% confidence interval [CI] 0.915–0.926), a sensitivity of 0.833 (95% CI 0.814–0.852) and a specificity of 0.851 (95% CI 0.848–0.855). Subsequent *p*‐values were derived from Mann–Whitney U tests comparing each model to this full multi‐omics model. The second‐highest sensitivity of 0.816 (95% CI 0.809–0.823, *p* = 0.241) was observed with phenomics (PHE), whereas the second‐highest specificity of 0.836 (95% CI 0.833–0.840, *p* = 0.0002) was achieved with the model trained on proteomics data (PRO). Furthermore, classifiers trained on medical history (HxQ), phenomics (PHE) and proteomics (PRO) exceeded a ROC AUC of 0.8, with ROC AUCs of 0.831 (95% CI 0.828–0.834, *p* = 0.00018), 0.862 (95% CI 0.860–0.865, *p* = 0.0002) and 0.899 (95% CI 0.895–0.903, *p* = 0.0002), respectively.

Additionally, a reduced multi‐omics model was developed using only 125 of the 3241 omics features selected through recursive feature elimination (RFE). No differences in performance over the full multi‐omics model were observed with this reduced model, as reflected in a ROC AUC of 0.923 (95% CI 0.920–0.927, *p* = 0.384), sensitivity of 0.839 (95% CI 0.827–0.850, *p* = 0.520) and specificity of 0.853 (95% CI 0.849–0.857, *p* = 0.909).

### Multicentre validation and feature importance analysis

All tuned models underwent out‐of‐sample validation in the pre‐specified cohort, comprising data from multiple centres. The best‐performing classifier configuration (LAB + PHE + MET + PRO) was selected for subsequent model validation and analysis of the importance of multi‐omics features. After excluding samples with more than 5% missing values, the final validation dataset, containing all of these omics, comprised 444 HFpEF patients and 6950 controls. Ten classifiers with different weight initializations were trained on the training cohort, achieving a ROC AUC of 0.931 (95% CI 0.930–0.931), a sensitivity of 0.857 (95% CI 0.855–0.860) and a specificity of 0.847 (95% CI 0.846–0.847) on the validation cohort. The full and reduced (RFE) multi‐omics models yielded comparable Brier scores of 0.110 (95% CI 0.109–0.112) and 0.108 (95% CI 0.106–0.111; *p* = 0.186), as well as classification accuracies of 0.850 (95% CI 0.847–0.854) and 0.852 (95% CI 0.848–0.856; *p* = 0.623), with consistent performance across additional evaluation metrics (*Figure* *2B*, online supplementary *Figure Appendix* *S1* and *Table* *S7*).

At the time of recruitment and multi‐omics data acquisition, 77.9% of individuals who subsequently developed HFpEF were asymptomatic. The classifier identified these asymptomatic patients as being at risk an average of 6.3 years (SD ± 3.9) before symptom onset (ROC AUC 0.929; 95% CI 0.916–0.942) (*Figure* *2C*). When applied to our multi‐stage selected patient cohort as an external, population‐based benchmark, the HFpEF‐ABA model yielded a ROC‐AUC of 0.835, comparable to the published reference model.^12^ The multi‐omics classifier exhibited improved performance relative to the HFpEF‐ABA model (*p* < 0.001; *Figure* *2D*).

The 30 omics features with the highest mean absolute SHAP values were identified. The following features were ranked in the top 30 for both the full multi‐omics (online supplementary *Figure* *S2A*) and RFE (online supplementary *Figure* *S2B*) models, highlighting their importance for model accuracy but not necessarily their pathophysiological relevance, as certain features could be recursively eliminated without significantly impacting model performance. Notably, greater age (>60 years), higher BMI (>30 kg/m^2^) and elevated levels of NT‐proBNP, NPPB, GDF15, REN, FABP3, CLSTN2, NPL and monocytes, along with lower levels of F10, FGFBP1, LRRN1, CCER2 and ADGRG2, remained as predictive indicators across both models. A separate SHAP analysis was conducted for the classifier trained solely on modifiable features (online supplementary *Figure* *S3*).

### Subgroup identification

To stratify a highly heterogeneous HFpEF cohort (*n* = 33 480) and identify more homogeneous subgroups, unsupervised SNF was applied, incorporating data from proteomics, phenomics, metabolomics and laboratory measurements. Only patients with data across all four data modalities were included, reducing the final sample size to 1646. Six distinct phenotype subgroups, each characterized by unique omics profiles, were identified through the analysis. Cluster 2 emerged as a high‐risk group with a mortality rate of 65.2% and a mean BMI of 34.9 kg/m^2^. Significant differences in basic demographic and health indicators among the clusters are summarized in online supplementary *Tables* *S8* and *S9*.

Clusters 3 and 5 were predominantly male (98.1% and 95.6%, respectively), with Cluster 5 having a lower average BMI (28.6 kg/m^2^) than Cluster 3 (34.3 kg/m^2^). Clusters 4 and 6 were primarily female (94.8% and 92.3%, respectively), with Cluster 6 showing the lowest average BMI (24.6 kg/m^2^) and the lowest mortality rate (14.7%) among all clusters, whereas Cluster 4 had a mean BMI of 34.3 kg/m^2^. Age at recruitment showed little variation between clusters, ranging from an average of 62.3 years (Cluster 3) to 64.8 years (Cluster 5).

Similarity network fusions enable quantification of the percentage of cases within each cluster that exhibit the highest similarity within specific omics modalities. This distribution is illustrated by upset plots (online supplementary *Figure* *S4*), where phenomics emerged as the predominant modality in Clusters 3 (39.7%), 4 (40.2%), 5 (29.2%) and 6 (53.7%). Cluster 1 exhibited the highest similarity within proteomics (47.1%). Notably, Cluster 2 was unique in demonstrating balanced similarity across all four omics dimensions, with contributions from phenomics (19.7%), proteomics (22.3%), laboratory measurements (20.2%) and metabolomics (19.9%). Kaplan–Meier survival curves for all sub‐clusters, diabetic controls and obese controls are shown in online supplementary *Figure* *S5* and the *Graphical Abstract*. Diabetic and obese controls had better survival estimates over a 15‐year follow‐up period compared to all HFpEF clusters.

### Subgroup characterization

To assess the distinctness of the six identified clusters within the broader UK Biobank population, the performance of two additional supervised CatBoost classifiers was evaluated using the five‐fold nested cross‐validation to predict cluster membership relative to the entire population. The summary of the performance metrics is presented in online supplementary *Table* *S9*.

The classifier trained on both omics and non‐omics features demonstrated superior ROC AUC across all clusters compared to the model trained exclusively on non‐omics features. The ROC AUC for the multi‐omics classifier ranged from 0.825 to 0.988, with sensitivity between 0.688 and 0.882, and specificity from 0.773 to 0.971. Notably, Cluster 2, which had the highest mortality rate, exhibited the best classifier performance (ROC AUC 0.988, 95% CI 0.985–0.990; specificity 0.971, 95% CI 0.965–0.977; sensitivity 0.882, 95% CI 0.788–0.941). The clinical non‐omics and omics features with the highest mean SHAP values are detailed in online supplementary *Table* *S9*.

The distinct molecular phenotype of Cluster 2, as identified by the highest mean SHAP values, was characterized by the up‐regulation of TNFRSF10B, TNFRSF1A, GDF15, EFNA4, ANGPT2, FABP4 and ITGBL1, alongside the down‐regulation of UMOD. Notably, questionnaires and patient history (non‐omics features) alone were sufficient to predict Cluster 2 cases, yielding a ROC AUC of 0.962 (95% CI 0.957–0.966), sensitivity of 0.855 (95% CI 0.823–0.892) and specificity of 0.926 (95% CI 0.920–0.931). This cluster was further associated with inactivity, higher BMI and comorbidities, including type 2 diabetes and chronic kidney disease.

### Confounder‐aware association analysis

**Figure 3:**
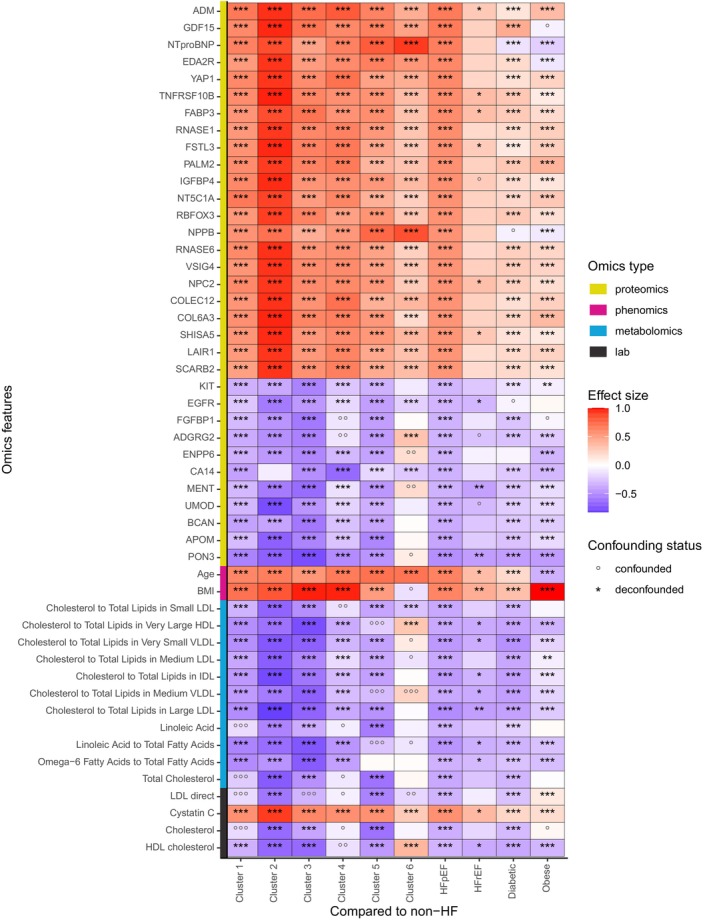
Confounding and deconfounding of multi‐omics associations within disease groups compared to controls. The heatmap displays the top multi‐omics associations (proteomics: yellow, phenomics: magenta, metabolomics: cyan, laboratory measurements: black) for various disease and control groups, including cases with at least 95% of multi‐omics features available. The groups analysed are: Cluster 1 (n_95_/n_all_: 211/268), Cluster 2 (170/204), Cluster 3 (252/309), Cluster 4 (282/346), Cluster 5 (302/363) and Cluster 6 (131/156), heart failure with preserved ejection fraction (HFpEF) (1646/33 480), heart failure with reduced ejection fraction (HFrEF) (34/486), diabetic controls (755/10 247) and obese controls (2549/38 772). All groups were compared to non‐heart failure (HF) controls (18 212/256 895). The strength of association, measured by Cliff's delta, is colour‐coded, with red and blue indicating positive and negative effect sizes, respectively. Each omics feature was tested for potential confounding variables: circles (o) denote confounding features and asterisks (*) indicate deconfounded features. The heatmap shows the top 25 features with the highest and lowest effect sizes for the HFpEF cohort. Statistical significance is indicated as follows: false discovery rate (FDR): *<0.1, **<0.01, ***<0.001, empty cells represent FDR ≥ 0.1.

The deconfounding analysis identified omics features significantly associated with various disease and control groups, independent of covariate effects, including medications, medical history and lifestyle factors derived from online questionnaires. This analysis encompassed the HFpEF population and its clusters, as well as groups with HFrEF, diabetes and obesity. The results were visualized in a heatmap (*Figure* *3*).

By comparing the HFpEF cohort to non‐HF controls, 2109 out of 3241 omics features, including laboratory measurements, phenomics, metabolomics and proteomics, were found to be associated with HFpEF, with an FDR <0.1. These features, which differentiated the diseased from non‐HF controls, were subsequently tested for confounding effects of other covariates. Of these, 1577 omics features were confidently deconfounded (their association could be attributed solely to disease status), 32 were ambiguously deconfounded (both the covariate and disease strongly correlated with the omics features) and 500 were confounded (the association between the disease and the omics features was confounded by at least one covariate).

*Figure* *3* presents the 25 omics features with the highest and lowest effect sizes (Cliff's delta) in the HFpEF population, accounting for potential confounding factors. The majority of both up‐regulated and down‐regulated features were proteomic markers. Among the up‐regulated proteins, the most prominent included ADM, GDF15, NTproBNP, EDA2R, YAP1, TNFRSF10B, FABP3, RNASE1, FSTL3, PALM2, IGFBP4, NT5C1A, RBFOX3, NPPB, RNASE6, VSIG4, NPC2, COLEC12, COL6A3, SHISA5, LAIR1 and SCARB2. The down‐regulated proteins predominantly consisted of PON3, APOM, BCAN, UMOD, MENT, CA14, ENPP6, ADGRG2, FGFBP1, EGFR and KIT. Cluster 2 exhibited the highest up‐regulation of most top proteins across all groups, except for NT‐proBNP and NPPB, which were most up‐regulated in Cluster 6. In contrast, the down‐regulated proteins showed more variability; only APOM, UMOD and EGFR were markedly decreased in Cluster 2. In terms of phenomics features, all clusters, except Cluster 6, demonstrated higher BMI compared to non‐HF controls. Moreover, only the obese control group was significantly younger than the non‐HF control group.

All top metabolomic features were down‐regulated across the majority of groups, except for Cluster 6. In Cluster 1, the observed reduction in total cholesterol levels was confounded by the use of cholesterol‐lowering and blood pressure medications. These medications, along with aspirin, were also confounding factors influencing linoleic acid levels in this cluster. In Cluster 5, cholesterol‐lowering therapies further contributed as confounders for the ratios of linoleic acid to total fatty acids, cholesterol to total lipids in medium very‐low‐density lipoprotein (VLDL) and cholesterol to total lipids in very large high‐density lipoprotein (HDL). In contrast, HDL cholesterol levels and the ratios of cholesterol to total lipids in medium VLDL and very large HDL were selectively elevated in Cluster 6, a group predominantly composed of female subjects. Across all clusters, the majority of laboratory measurements demonstrated a consistent trend of down‐regulation, except for cystatin C, which was elevated in HFpEF patients, particularly in Cluster 2. Additionally, online supplementary *Figure* *S6* underscores the key omics features characterizing each cluster, as identified from the most important features listed in online supplementary *Table* *S9*. These features were derived from a supervised cluster classifier trained to distinguish cases within each cluster from those not assigned to the cluster.

### Pathway analysis

**Figure 4:**
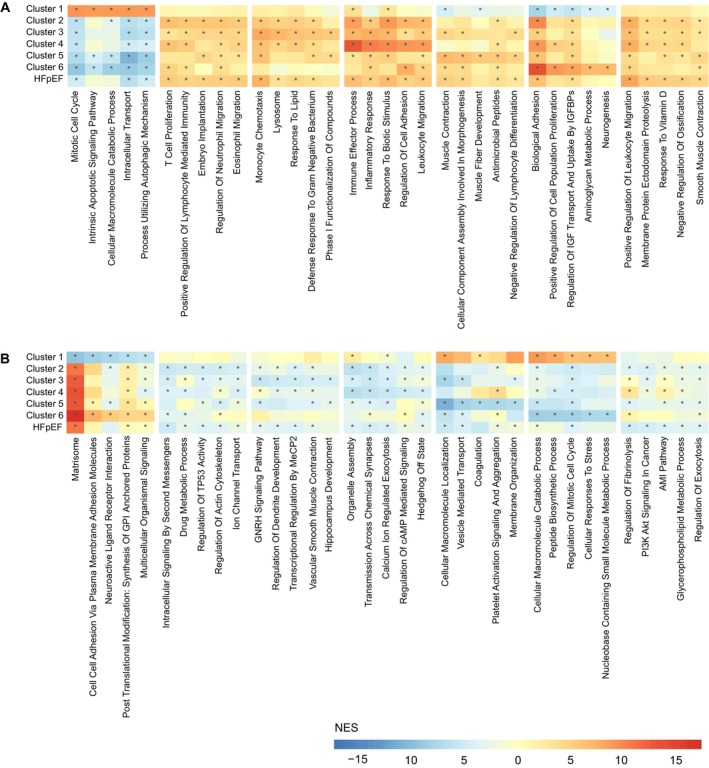
Disease group‐specific pathway activity from single‐sample gene set enrichment analysis (ssGSEA). Heatmaps displaying normalized enrichment scores (NES) for the top five upregulated (*A*) and downregulated (*B*) pathways across clusters 1–6 with the furthest right block representing the most significant pathways within the entire heart failure with preserved ejection fraction (HFpEF) group. The input for ssGSEA consisted of effect sizes from the confounding analysis of proteomic measurements, where each group was compared to healthy controls. Pathways were considered cluster‐specific if they had an enrichment score of at least one standard deviation from the mean and exhibited the maximum (*A*) or minimum (*B*) value for that pathway within the cluster. The heatmaps highlight significant changes in pathway activity across disease groups, using gene ontology biological pathways and curated pathways from the molecular signature database (MSigDB). *Group‐specific FDR <0.05.

To further elucidate biological differences both between the HFpEF clusters and at the cohort level, ssGSEA was performed using the calculated effect sizes of proteomics data in disease groups compared to non‐HF controls. Cluster‐specific pathways were identified using an FDR cut‐off of 0.05 and an enrichment score corresponding to the maximum or minimum value within each cluster. *Figure* *4* presents the five highest‐ and lowest‐scoring cluster‐specific pathways.

In the entire HFpEF cohort, there was an up‐regulation of immune‐related pathways. Cluster 1 exhibited up‐regulation in the cell cycle and apoptosis pathways, along with a depletion of extracellular matrix (ECM) proteins. Cluster 2 showed down‐regulation of drug metabolic processes and ion transport channels, coupled with increased cellular immune responses. Cluster 3 pathways were predominantly related to metabolic dysregulation and involved proteins associated with bone formation. Cluster 4 demonstrated a broad increase in immune and inflammatory pathways without a specific focus on cellular immune responses. Cluster 5 featured pathways associated with muscle function and development. Cluster 6 displayed significant alterations in biological adhesion pathways, an increase in ECM proteins, metabolic dysregulation and a reduced oxidative stress response.

## Discussion

Our multi‐omics machine learning classifier demonstrated high accuracy in identifying HFpEF‐related phenotypes during predominantly asymptomatic stages, thereby underscoring its potential for early and reliable disease detection. Multicentre validation confirmed the model's reproducibility and performance consistency. Additionally, SNF clustering revealed a distinct subgroup characterized by high mortality (65.2%), associated with comorbidities such as obesity, type 2 diabetes and chronic kidney disease. The molecular phenotype of this high‐risk group indicates dysregulation of inflammatory signaling pathways, consistent with current concepts of metabolic inflammation in HFpEF.^32^

### Implications for heart failure with preserved ejection fraction screening strategies

A recent evaluation of the HFpEF‐ABA score for screening HFpEF in patients with dyspnoea demonstrated good discrimination (ROC AUC 0.840 for the reference model).^12^ Applying the HFpEF‐ABA reference model to the UK Biobank cohort yielded similar performance (ROC AUC 0.835), confirming the reliability of our labeling and patient selection algorithm as a basis for classifier training. While the HFpEF‐ABA model provides a basic screening tool for symptomatic patients with dyspnoea, our multi‐omics classifier identified patients at risk of HFpEF pre‐clinically—on average, 6.3 years before symptom onset—in a large, predominantly asymptomatic, multicentre cohort (ROC AUC 0.931; 95% CI 0.930–0.931).

Despite the high diagnostic performance of multi‐omics approaches, cost‐effective methods, such as phenomics and questionnaire‐based assessments, remain indispensable for large‐scale screening and initial evaluations. In our study, a questionnaire‐based classifier performed comparably to established scoring models when using similar input features (ROC AUC 0.829; 95% CI 0.829–0.829). In clinical practice, these approaches could complement each other within a staged detection strategy, enabling broader population‐level screening in resource‐limited settings, followed by targeted multi‐omics analyses for patients at increased risk, with embedded individual SHAP explanations. Of note, a fully analytically validated multi‐protein serum assay has recently become available for clinical research in multiple sclerosis and has been developed to facilitate future implementation in clinical decision‐making.^33^

### Confounder‐aware disease associations and subgroup‐specific profiles

To enhance interpretability and further ensure the robustness of our findings, we employed XAI alongside a rigorous deconfounding approach^17^ to account for potential biasing factors. Using nested post‐hoc model comparison tests, the confounder‐aware association analysis provided a framework to distinguish genuine disease associations from confounding‐driven effects, thereby highlighting how anthropometrics, comorbidities, environmental exposures, medication use and lifestyle factors can introduce bias or modulate omics‐based disease associations. Lipidomic profiles across clusters were shaped by medication‐related confounding and sex‐specific modulation, whereas many other omics features remained robust after adjustment for confounding factors.

It is known from transcriptomic analyses of invasive endomyocardial biopsies that HFpEF comprises distinct molecular subgroups associated with specific clinical features and outcomes and is frequently accompanied by comorbidities, particularly metabolic disorders such as obesity and diabetes.^34^ When these comorbidities co‐exist with HFpEF, patients exhibit higher mortality and significantly worse survival outcomes over the 15‐year follow‐up compared to those with obesity or diabetes alone (online supplementary *Figure* *S5*). This suggests that HFpEF introduces distinct pathophysiological mechanisms that exacerbate prognosis, compounding the impact of common comorbidities like obesity and diabetes.

The molecular changes found were dominated by markers of systemic inflammation, haemodynamic stress, renal impairment, and endothelial dysfunction, mirroring the immunometabolic crosstalk recently summarized by the ESC Heart Failure Association.^35^ Although cluster analysis depends on selected cohorts and feature sets, offering relative rather than absolute classification, identifying patients belonging to the high‐risk subgroup (Cluster 2) may facilitate recognition of individuals prone to adverse outcomes. In this cluster, elevated pro‐inflammatory markers, particularly TNFRSF1A, a mediator of tumour necrosis factor‐α‐driven vascular inflammation, suggest a role for chronic inflammatory stress. Similarly, growth differentiation factor‐15 has emerged as a prognostic biomarker in heart failure, associated with oxidative stress, inflammation, and adverse outcomes.^36^ Its elevation across all clusters supports the central role of inflammation. The increased expression of apoptosis‐inducing receptors TNFRSF10A (DR4) and TNFRSF10B (DR5) indicates activation of cell death pathways, potentially reflecting endothelial cell injury. This aligns with the non‐ischaemic, microvascular dysfunction that characterizes HFpEF.

Furthermore, up‐regulation of angiogenesis‐related factors, including ANGPT2 and EFNA4, points to vascular remodeling. ANGPT2 destabilizes endothelial integrity and promotes inflammation‐induced vascular leakage, while EFNA4 is involved in endothelial adhesion and vessel pruning. Their dysregulation may contribute to the abnormal capillary density and perfusion deficits characteristic of HFpEF. Furthermore, the higher levels of ADM, a vasoactive peptide linked to haemodynamic stress together with reduced UMOD, a marker indicative of renal tubular impairment, support a cardio‐renal continuum. Finally, increased levels of YAP1, a transcriptional co‐activator in the Hippo signaling pathway, suggest altered mechanotransduction and endothelial cell behaviour, potentially modulating fibrosis, apoptosis, and angiogenesis in response to mechanical and metabolic stress. These biomarkers represent potential targets for preventive strategies.

### Signaling pathway activity

While more in‐depth investigations are needed to fully elucidate the pathophysiological impact of the different molecular factors, the ssGSEA analysis of pathway activity provides valuable initial insights and supports the generation of future research hypotheses. The up‐regulation of inflammatory pathways in the entire HFpEF cohort reinforces the concept of ‘metabolic inflammation’ as a key component in HFpEF pathophysiology.^32^ However, markedly exaggerated immune responses in Clusters 2 and 4, with pronounced up‐regulation of cellular immune pathways in Cluster 2 and immune effector processes and innate inflammatory responses in Cluster 4, strongly indicate differences in underlying mechanisms, and correlate with notable clinical comorbidity burdens (online supplementary *Table* *S8* and *Figure* *S7*). Additionally, the down‐regulation of drug metabolic processes in Cluster 2 may be associated with compromised liver function, potentially affecting medication clearance. The increased expression of biological adhesion and ECM proteins across all subgroups, most pronounced in Cluster 6, may point towards a common role for fibrosis and tissue remodeling. Collectively, these findings highlight the heterogeneity of HFpEF, alongside the need for proactive management of comorbidities.

### Limitations

The absence of a specific diagnostic label for HFpEF within the UK Biobank constitutes an acknowledged limitation. To address this, we applied established clinical guideline criteria and literature‐supported evidence for case identification. Our approach aimed to mirror the diagnostic processes used in clinical practice, reflecting the inherent challenges of identifying HFpEF in routine clinical settings, where scoring systems can support diagnosis and typically align with confirmed disease classification.^11^, ^23^ The robustness of this patient selection algorithm is further supported by its comparable performance when applying the HFpEF‐ABA reference model^12^ to our cohort selection and alignment with previously reported HFpEF prevalence in a comparable age range.^1^ Due to the absence of absolute NT‐proBNP concentrations, our selection approach using the 90th percentile differs from guideline‐based absolute cut‐offs but remains conceptually aligned, as similarly reported by a previous population‐based study,^37^ in which 3–8% of men and 15–20% of women aged 40–59 years exceeded the guideline‐recommended NT‐proBNP threshold of 125 pg/ml. Furthermore, the temporal limitation of single time point clustering precludes assessing whether pre‐HFpEF patients consistently cluster into stable groups or diverge into distinct trajectories as clinical symptoms develop over time. Furthermore, despite the application of rigorous deconfounding methods, the potential influence of unmeasured confounders cannot be entirely excluded.

Additionally, the study's reliance on a predominantly European cohort limits the generalizability of the findings to more diverse global populations.^38^ Moreover, the reduction in sample size due to the requirement of having all data modalities available posed a challenge for model training, particularly for models containing proteomics data (*n* < 30 000) compared to those using modalities available for all patients (*n* > 300 000). To allow fair comparison between models, in addition to validation using the entire validation cohort (*n* = 100 446), we performed model comparisons on the subset of patients in the validation cohort where all modalities were available (*n* = 7394).

While the Olink blood proteomics panels, which utilize proximity extension assay technology, provide a robust approach to protein quantification, the relative nature of NPX units limits direct comparison of absolute protein levels across studies. Implementation strategies can include the development of custom panels for absolute quantification of specific proteins to further support clinical translation.^33^ Alternatively, selected targets or reduced models can be reformatted for use with immunoassay platforms that allow absolute quantification, such as enzyme‐linked immunosorbent assays.

## Conclusions

A multi‐omics‐based machine learning framework enables early identification of HFpEF during pre‐clinical stages, when timely interventions may still modify disease progression. This approach also facilitates the molecular characterization of at‐risk individuals, thereby enhancing risk stratification and supporting targeted treatment strategies. Comprehensive molecular profiling and pathway‐level analyses revealed distinct inflammatory signaling patterns, along with evidence of fibrosis and tissue remodeling, partially elucidating the heterogeneity inherent to HFpEF. These findings support the conceptualization of HFpEF as a metabolic‐inflammatory disorder and underscore the translational potential of large‐scale multi‐omics studies for pre‐clinical disease detection and mechanistic insights into complex cardiovascular phenotypes.

## Supporting information


**Appendix S1.** Supporting Information.
