# Supplementary material for: Deep phenotyping of heart failure with preserved ejection fraction through multi‐omics integration
Source: Eur J Heart Fail. 2025 Sep 22;27(12):3243–59. doi: 10.1002/ejhf.70041 (PMC12803610; doi:10.1002/ejhf.70041)
Supplement: Supplementary file 1 — Appendix S1. Supporting Information. [file EJHF-27-3243-s001.pdf]

Supplementary Material

Deep Phenotyping of Heart Failure with Preserved Ejection Fraction through Multi-Omics Integration

Jakob Versnjak, Titus Kuehne, Pauline Fahjen, Nina Jovanovic, Ulrike Löber, Gabriele G. Schiattarella, Nicola Wilck, Holger Gerhardt, Dominik N. Müller, Frank Edelmann, Philipp Mertins, Roland Eils, Michael Gotthardt, Sofia K. Forslund, Benjamin Wild, Marcus Kelm

Supplementary Methods

Supplementary Tables

Supplementary Table 1: Summary of HFpEF selection criteria

Supplementary Table 2: Clinical features and corresponding phecodes

Supplementary Table 3: Clinical features and corresponding OMOP concepts

Supplementary Table 4: Medications and corresponding OMOP concepts

Supplementary Table 5: Extended baseline characteristics of the HFpEF cohort and control groups

Supplementary Table 6: Performance evaluation of nested 10-fold cross-validation on the training cohort for different modality combinations

Supplementary Table 7: Performance evaluation of classifiers validated on the validation cohort for different modality combinations

Supplementary Table 8: Extended characteristics of the HFpEF clusters and control groups

Supplementary Table 9: Characteristics and top features of HFpEF clusters

Supplementary Figures

Supplementary Figure 1: Performance metrics on the expanded validation cohort

Supplementary Figure 2: SHAP analysis of full and reduced multi-omics classifiers

Supplementary Figure 3: Performance and SHAP analysis of classifier trained on modifiable features

Supplementary Figure 4: Upset plots illustrating similarity overlap among affinity matrices prior to SNF

Supplementary Figure 5: Kaplan–Meier survival estimates comparing HFpEF clusters and control groups

Supplementary Figure 6: Confounding and deconfounding of multi-omics associations with clusters compared to healthy controls

Supplementary Figure 7: Comorbidities and medication use across HFpEF clusters

Supplementary Methods

Study participants

Symptoms shown in Table S1 were considered, however, they were never used in isolation for classification. Our symptom-based identification approach incorporated all typical symptoms recommended by ESC guidelines (breathlessness/dyspnoea, orthopnoea, paroxysmal nocturnal dyspnoea, reduced exercise tolerance, fatigue excluding post-viral fatigue, ankle swelling), as well as syncope and atrial fibrillation, indicative of implicit clinical signs/symptoms like palpitations, tachycardia, dyspnoea, exertional intolerance and fatigue.[1] Subsequently, more than 99.7% of all patients classified as HFpEF either exhibited or developed symptoms listed or implied by the ESC diagnostic criteria, or had a heart failure diagnosis code (Table S1). Additionally, nocturia was included as a documented symptom in stable and acute heart failure due to its plausibility related to nocturnal natriuretic peptide-mediated diuresis.[2] In the UK Biobank cohort, nocturia was typically accompanied by additional heart failure symptoms and was the sole presenting symptom in only 94 individuals (0.28%). Exclusion of these cases led to a marginal reduction in ROC-AUC, decreasing from 0.9320 to 0.9310 (95% CI 0.9301–0.9317), corresponding to a negligible difference of $\Delta = 0.001$.

Further sensitivity analyses were performed to confirm the robustness and stability of the model's predictive performance. We conducted a sensitivity analysis using a stricter 95th-percentile NPX cut-off for NT-proBNP (instead of the 90th percentile). The classifier performance remained highly consistent, demonstrating negligible impact of varying this threshold: ROC-AUC = 0.9285 (95% CI: 0.9281–0.9292). When cases in which syncope was the sole additional symptom were excluded in a sensitivity analysis, the size of the cohort was reduced from 33,480 to 33,035 (-1%). The corresponding change in the ROC AUC was minimal, from 0.9310 (0.9300–0.9319) to 0.9302 (0.9287–0.9316, $\Delta = 0.0008$), indicating a negligible impact on model performance for this less typical clinical symptom. A targeted clinical audit of the final OMOP/PheCode and ICD-10 concept selections, as well as symptom mappings, was performed by three specialists with recognised expertise in cardiovascular disease and heart failure.

As the HFA-PEFF score could not be implemented owing to the unavailable required parameters. An H_2FPEF-guided diagnostic algorithm, supplemented by clinical symptoms, NT-proBNP measurements, and diagnostic codes, was employed for HFpEF case selection. To provide an external population-based benchmark, the performance of the published HFpEF-ABA reference model was evaluated in cases identified through this multi-stage selection process and subsequently compared with previously published data.

Isolated 'diastolic HF' codes showed high PPV but modest sensitivity in HFpEF identification.[3] To increase specificity, we required the presence of concordant heart failure symptoms and, for other heart failure diagnostic codes, additional biomarker or imaging evidence, in line with recent validation studies.[3]

Data modalities

A 10-fold cross-validation experiment was conducted using a logistic classifier from the scikit-learn Python package (v1.4.1) with default hyperparameters. For each fold, the top 100 SNPs with the highest absolute coefficient values were identified, and only SNPs consistently ranked in the top 100 across all folds were included in the genomics dataset.

Machine learning approaches

Affinity matrices for each data modality were constructed using the Gaussian kernel with Euclidean distance, with missing values excluded from Euclidean distance computation. Although the SNF

supports missing data, cases containing more than 30% missing values in any omics modality were removed. The four affinity matrices were then combined using an iterative algorithm to produce a fused affinity matrix, which was subsequently subjected to spectral clustering to identify distinct phenotype groups. The SNF method was adapted in Python. The optimal number of clusters was determined using the eigengap heuristic method, complemented by the distinct separation of clusters based on patient demographics and clinical characteristics, including sex and mortality rate, ensuring biological and clinical relevance.[4,5]

Samples with more than 5% missing data were excluded to minimise the predictive effects of missing values. Categorical variables were transformed into a binary format using one-hot encoding, and continuous features were normalised to a range between 0 and 1. Hyperparameters such as learning rate (0.01, 0.05, 0.1), L2 regularisation strength (1, 3, 5), boosting type (ordered, plain) and column sampling levels (0.8, 0.9, 1.0) were tuned using grid search. To further reduce the risk of overfitting, the number of iterations (100) and tree depth (3) were deliberately kept low. To address data imbalance, weights were adjusted based on the disease-control ratio, and the area under the receiver operating characteristic curve (ROC AUC) was used as the loss function. The outer cross-validation fold was used to evaluate the performance of fine-tuned models, with model evaluation metrics including ROC AUC, accuracy, sensitivity ((True positives)/(True positives + False negatives)) and specificity ((True negatives)/(True negatives + False positives)). To identify the subset of the most important multi-omics features, recursive feature elimination (RFE) with 10-fold cross-validation was applied using the scikit-learn library.

We evaluated Bayesian optimisation (Tree-structured Parzen Estimator, 50 trials) against our original grid-search strategy for the LAB + PHE + MET + PRO CatBoost model. Bayesian optimisation improved median ROC AUC by only 0.01 percentage points (two-sided Mann–Whitney U, $p = 0.969$) while increasing computation time from 2 h to 16 h per model. Given the negligible benefit and substantially higher cost, grid search was retained for all analyses.

Furthermore, the control group was stratified by sex to match the distribution in the clusters, ensuring that the features were not confounded, as many clusters predominantly consisted of a single sex. These classifiers then underwent the nested 5-fold cross-validation. Two sets of classifiers were trained. The first classifier aimed to identify distinct clinical features characterising each cluster by using only non-omics features not included in the unsupervised clustering, such as medications, diagnoses and lifestyle factors. Cut-off values for these features were determined based on dependence plots, with only those features selected that consistently ranked among the 15 highest mean SHAP values across all folds. The second classifier was trained using both non-omics and omics features to discern specific multi-omics profiles for each cluster.

References

1. Ko D, Chung MK, Evans PT, Benjamin EJ, Helm RH. Atrial Fibrillation: A Review. *JAMA* 2025;**333**:329-342. doi: 10.1001/jama.2024.22451
2. Ohishi M, Kubozono T, Higuchi K, Akasaki Y. Hypertension, cardiovascular disease, and nocturia: a systematic review of the pathophysiological mechanisms. *Hypertens Res* 2021;**44**:733-739. doi: 10.1038/s41440-021-00634-0
3. Cohen SS, Roger VL, Weston SA*, et al*. Evaluation of claims-based computable phenotypes to identify heart failure patients with preserved ejection fraction. *Pharmacol Res Perspect* 2020;**8**:e00676. doi: 10.1002/prp2.676
4. von Luxburg U. A tutorial on spectral clustering. *Statistics and Computing* 2007;**17**:395-416. doi: 10.1007/s11222-007-9033-z
5. Al-Zaiti SS, Suba S, Carey MG. Challenges Associated With Waveform Morphology Interpretation of 12-Lead Electrocardiograms. *Am J Crit Care* 2023;**32**:463-464. doi: 10.4037/ajcc2023896

Supplementary Table 1: Summary of HFpEF selection criteria

A summary of features included in the selection criteria for identifying HFpEF patients, with the number of cases (n) satisfying each criterion (rows 1-5). Row 6 explains how the criteria from rows 1-5 were combined to identify the HFpEF cohort.

**Selection criteria**			n=
1		**Diagnostic coding**	
	a	Heart failure	22,225
	b	Diastolic heart failure	169
2		**CMR-based imaging information**	
	a	Ejection fraction available	40,302
	b	Ejection fraction available and normal	39,110
3		**Biomarker**	
		NT-proBNP > 90th percentile	4,966
4		**Scoring**	
	a	HFpEF pretest risk > 90%	13,997
	b	HFpEF pretest risk > 70%	63,394
5		**Clinical symptoms/signs**	
	a	Dyspnoea/Orthopnoea	42,902
	b	Ankle/leg oedema	9,335
	c	General/local/unspecified oedema	7,852
	d	Nocturia	4,797
	e	Fatigue without post-infectious fatigue	38,620
	f	Nocturnal dyspnoea	200
	g	Syncope	26,743
	h	Atrial fibrillation with HFpEF pretest risk > 80%	22,467
	i	Heart failure	22,225
6	a	(1 AND NOT 2b) AND 5	635
	b	NOT 6a AND 2b AND (3 OR 4b OR 1) AND 5	919
	c	NOT 6a AND NOT 6b AND (4a AND 2b) AND 5	11,778
	d	NOT 6a AND NOT 6b AND NOT 6c AND (4b AND (3 OR 1 OR 2b)) AND 5	20,148
		6a OR 6b OR 6c OR 6d	33,480

Supplementary Table 2: Clinical features and corresponding phecodes

This table lists clinical conditions (features) alongside their corresponding phecodes used for phenotyping. Some features are associated with multiple phecodes. *Clinical features and diagnoses employed for diagnostic coding or characterisation of heart failure presentations.

Feature name	Phecode
Postviral fatigue* (exclusion criterion)	807.11
Dyspnoea*	488.1
Diastolic heart failure*	424.3
Heart failure*	424
Left heart failure*	424.1
Malaise and fatigue*	807
Nocturia*	594.41
Syncope and collapse*	808
Type 2 diabetes	202.2
Myocardial infarction	404.11, 404.1
Endocarditis	410.2
Stroke and transient cerebral ischemic attacks	431, 431.1, 431.11, 431.12, 431.2, 431.15, 431.14, 431.13
Epilepsy, recurrent seizures, convulsions	330, 330.1, 330.11, 330.12, 330.3, 330.31, 330.13
Venous thromboembolism	440.1
Embolism and thrombosis	440
Phlebitis and thrombophlebitis	440.13
Pulmonary embolism	440.3
Arterial embolism and thrombosis	440.2
Deep vein thrombosis [DVT]	440.11

Supplementary Table 3: Clinical features and corresponding OMOP concepts

This table lists clinical features alongside their corresponding OMOP concepts. The "Feature name" column presents the clinical conditions of interest, while the "OMOP Concepts" column includes the standardised OMOP vocabulary terms mapped to each feature. *Clinical features and diagnoses employed for diagnostic coding or characterisation of heart failure presentations.

Feature name	OMOP Concepts
Heart failure with normal ejection fraction*	['Heart failure with normal ejection fraction']
Hypertensive heart disease with congestive heart failure*	['Hypertensive heart disease with congestive heart failure']
Nocturnal dyspnoea*	['Paroxysmal nocturnal dyspnoea', 'Nocturnal dyspnoea']
Fatigue*	['Fatigue', 'Chronic fatigue syndrome', 'Malaise and fatigue', 'Exhaustion', 'Exhaustion due to exposure', 'Exhaustion - physiological', 'Exhaustion due to excessive exertion', 'Exhaustion during labor', 'Lethargy', 'Gets drowsiness', 'Intermittent drowsiness', 'Weakness present', 'Physical AND emotional exhaustion state', 'Quickly exhausted', 'O/E - drowsy'
Postviral fatigue syndrome* (exclusion criterion)	['Postviral fatigue syndrome']
Dyspnoea*	['Dyspnea', 'Dyspnea on exertion', 'Dyspnea at rest', 'O/E - dyspnea', 'Borg Breathlessness Score: somewhat severe', 'Borg Breathlessness Score: severe', 'Borg Breathlessness Score: very severe', 'Increasing breathlessness', 'Difficulty breathing', 'Labored breathing', 'Abnormal breathing', 'Respiratory distress', 'O/E - air hunger', 'Wheezing symptom', 'Gasping for breath', 'Breathing aggravates symptom', 'Interrupted breathing', 'Intense breathing', 'Increased breath sounds', 'Unable to breathe', 'Catching breath', 'Difficulty controlling breathing', 'Unable to complete a sentence in one breath']
Nocturia*	['Nocturia']
Swollen ankles*	['O/E - edema of ankles', 'Ankle edema', 'Worsening peripheral oedema', 'Peripheral edema', 'Dependent edema', 'Gravitational edema of leg', 'Leg swelling symptom', 'Foot swelling', 'Toe swelling']
Heart failure*	['Suspected heart failure', 'Left heart failure', 'Heart failure', 'Congestive heart failure', 'Congestive heart failure due to valvular disease', 'Congestive heart failure monitoring', 'Hypertensive heart disease with congestive heart failure', 'Biventricular congestive heart failure', 'Hypertensive heart and renal disease with both (congestive) heart failure and renal failure', 'Acute heart failure', 'Acute congestive heart failure', 'Chronic congestive heart failure', 'Hypertensive heart and renal disease with (congestive) heart failure'
Syncope*	['Syncope', 'Syncope and collapse', 'Vasovagal syncope', 'Syncope symptom', 'O/E - collapse - syncope', 'Micturition syncope', 'Tussive syncope', 'Situational syncope']
Impaired exercise tolerance*	['Exercise tolerance test abnormal', 'Avoids even trivial exercise', 'Impaired exercise tolerance', 'Exercise aggravates symptom', 'Worsening exercise tolerance']
Orthopnoea*	['Orthopnea', 'O/E - orthopnea']
Acute renal failure	['Acute renal failure syndrome', 'Acute renal failure due to crush syndrome', 'Hypertensive renal failure', 'Acute renal failure due to tubular necrosis', 'Postpartum acute renal failure', 'Acute renal failure due to acute cortical necrosis', 'Postoperative renal failure', 'Acute renal papillary necrosis with renal failure', 'Acute drug-induced renal failure', 'Prerenal renal failure', 'Transient acute renal failure', 'Acute renal failure due to ACE inhibitor', 'Nephrotoxic acute renal failure', 'Acute renal failure due to obstruction']
Chronic kidney disease	['Chronic kidney disease stage 3', 'Chronic kidney disease', 'Chronic kidney disease stage 3B without proteinuria', 'Chronic kidney disease stage 5', 'Chronic kidney disease stage 3 without proteinuria', 'Chronic kidney disease stage 4', 'Chronic kidney disease stage 5 without proteinuria', 'Chronic kidney disease stage 4 with proteinuria', 'Chronic kidney disease stage 3A', 'Chronic kidney disease stage 2', 'Chronic kidney disease stage 1', 'Chronic kidney disease stage 2 with proteinuria', 'Chronic kidney disease stage 3 with proteinuria', 'Chronic kidney disease stage 3A without proteinuria', 'Chronic kidney disease stage 3B with proteinuria', 'Chronic kidney disease stage 3B', 'Chronic kidney disease stage 5 with proteinuria', 'Chronic kidney disease stage 4 without proteinuria', 'Chronic kidney disease stage 2 without proteinuria', 'Chronic kidney disease stage 3A with proteinuria', 'Chronic kidney disease stage 1 without proteinuria', 'Chronic kidney disease stage 1 with proteinuria', 'Chronic kidney disease laboratory study']
Hospital admission	['Emergency hospital admission', 'Non-urgent hospital admission', 'Diabetic emergency hospital admission', 'Neurosurgical emergency hospital admission', 'Neurology emergency hospital admission', 'Rheumatology emergency hospital admission', 'Hematology emergency hospital admission', 'Geriatric emergency hospital admission', 'Cardiothoracic emergency hospital admission', 'Renal medicine emergency hospital admission', 'Cardiology emergency hospital admission', 'Emergency hospital admission for chronic obstructive pulmonary disease', 'Respiratory emergency hospital admission', 'Emergency hospital admission for heart failure', 'Vascular surgery emergency hospital admission', 'Emergency hospital admission for ischemic heart disease', 'Under care of hospital admission prevention service']
Myocardial infarction	['Silent myocardial infarction', 'Acute myocardial infarction', 'Acute myocardial infarction of inferior wall', 'Acute ST segment elevation myocardial infarction', 'EKG: myocardial infarction', 'Acute myocardial infarction of anterior wall', 'Acute myocardial infarction of inferolateral wall', 'Acute anteroseptal myocardial infarction', 'Acute myocardial infarction of anterolateral wall', 'Acute posterior myocardial infarction', 'Coronary thrombosis not resulting in myocardial infarction', 'Acute myocardial infarction of lateral wall', 'Acute myocardial infarction of inferoposterior wall', 'Acute non-ST segment elevation myocardial infarction', 'Acute anteroapical myocardial infarction', 'Postoperative myocardial infarction', 'Subsequent myocardial infarction', 'Postoperative subendocardial myocardial infarction', 'First myocardial infarction', 'Subsequent myocardial infarction of inferior wall', 'Subsequent myocardial infarction of anterior wall', 'Non-Q wave myocardial infarction', 'True posterior myocardial infarction', 'Acute myocardial infarction of posterolateral wall', 'Acute Q wave myocardial infarction', 'Acute myocardial infarction of septum', 'Acute myocardial infarction of atrium', 'Acute widespread myocardial infarction']

Endocarditis	['Acute rheumatic endocarditis', 'Subacute bacterial endocarditis', 'Acute bacterial endocarditis', 'Valvular endocarditis', 'Acute and subacute endocarditis', 'Bacterial endocarditis', 'Acute and subacute bacterial endocarditis', 'Rheumatic endocarditis', 'Candidal endocarditis', 'Infective endocarditis', 'Acute endocarditis', 'Prosthetic valve endocarditis', 'At risk of endocarditis']
Seizure	['Seizure', 'Epileptic seizure', 'Tonic-clonic seizure', 'Generalized seizure', 'Epileptic seizures - tonic', 'Epileptic seizures - clonic', 'Complex partial epileptic seizure', 'Witnessed epileptic seizure', 'Reflex anoxic seizure', 'Myoclonic seizure', 'Epileptic seizures - atonic', 'Atonic seizure', 'Daily seizures', 'Complex partial seizure with impairment of consciousness', 'Localization-related(focal)(partial)idiopathic epilepsy and epileptic syndromes with seizures of localized onset', 'Trigger factor for seizure', 'Seizure related finding', 'Partial seizure', '2 to 4 seizures a month', 'Simple partial epileptic seizure', '1 to 12 seizures a year', 'Situation-related seizures', '1 to 7 seizures a week', 'Many seizures a day', 'Isolated seizures', 'Epileptic seizures - akinetic', 'Absence seizure with impairment of consciousness only', 'Tonic seizure', 'Simple partial seizure with focal motor signs without march', 'Absence seizure with automatisms', 'Simple partial seizure with somatosensory or special sensory dysfunction', 'Visual seizure', 'Seizure undetermined whether focal or generalized', 'Clonic seizure', 'Gelastic seizure', 'Affective seizure', 'Dysphasic seizure']
Swelling of joints	['Soft tissue swelling of ankle joint', 'Bony swelling of ankle joint', 'Ankle joint synovial swelling', 'Foot joint swelling', 'Foot joint', 'Bony swelling of the foot joint']
Multivalve diseases	['Diseases of mitral and aortic valves', 'Disorders of both mitral and tricuspid valves', 'Combined disorders of mitral, aortic and tricuspid valves', 'Disorders of both aortic and tricuspid valves', 'Multiple valve disease']

Supplementary Table 4: Medications and corresponding OMOP concepts

This table lists medications of interest in the "Medication Name" column, alongside their corresponding OMOP concepts in the "OMOP Concepts" column.

**Medication Name**	**OMOP Concepts**
Beta blocker	sotalol, atenolol, bisoprolol, carvedilol, labetalol, metoprolol, propranolol
ACE-inhibitor	captopril, cilazapril, enalapril, fosinopril, imidapril, lisinopril, moexipril, perindopril, quinapril, ramipril, trandolapril
ARB	irbesartan, valsartan, losartan, candesartan, telmisartan, sacubitril
Mineralocorticoid receptor antagonist	spironolactone, eplerenone
Dapagliflozin	empagliflozin, dapagliflozin
Loop diuretics	furosemide
Levothyroxine	levothyroxine
Metformine	metformine
Thiazide diuretics	metolazone, hydrochlorothiazide, hydrochlorothiazide, bendroflumethiazide, hydrochlorothiazide, hydroflumethiazide, polythiazide, hydrochlorothiazide, cyclopenthiazide, hydrochlorothiazide
Ca-channel blocker	amlodipine, diltiazem, verapamil, nifedipine, nicardipine, felodipine, isradipine, nisoldipine, lacidipine, nimodipine, lercanidipine
Warfarin	warfarin
Aspirin	aspirin
Sulfonylureas	glipizide, gliclazide, glimepiride, tolbutamide, chlorpropamide, tolazamide
Iron therapy	ferrous sulfate, ferrous gluconate, ferrous fumarate, iron sucrose

Supplementary Table 5: Extended baseline characteristics of the HFpEF cohort and control groups

Comparison of baseline characteristics (extended Table 1) between non-HF controls (n = 256,895), obese controls (n = 38,772), diabetic controls (n = 10,247), and HFpEF (n = 33,480). Variables are presented as mean with standard deviation in the brackets. Differences in continuous variables between groups were analysed using either linear regression main effects testing or the Kruskal–Wallis rank test. Categorical variables were compared using Pearson's χ^2 test or Fisher's exact test, as appropriate.

	Non-HF controls (n = 256,895)	Obese controls (n = 38,772)	Diabetic controls (n = 10,247)	HFpEF (n = 33,480)	p-value
Assessment centre					
Stockport (pilot)	2,190 (0.9%)	363 (0.9%)	137 (1.3%)	252 (0.8%)	<0.001
Manchester	7,541 (2.9%)	1,364 (3.5%)	396 (3.8%)	970 (2.9%)	
Oxford	8,852 (3.4%)	1,085 (2.8%)	261 (2.5%)	759 (2.3%)	
Cardiff	7,253 (2.8%)	1,265 (3.3%)	247 (2.4%)	1,428 (4.3%)	
Glasgow	9,147 (3.6%)	1,484 (3.8%)	202 (1.9%)	1,269 (3.8%)	
Edinburgh	10,233 (4.0%)	1,324 (3.4%)	172 (1.6%)	853 (2.5%)	
Stoke	10,149 (4.0%)	1,852 (4.8%)	447 (4.3%)	1,401 (4.2%)	
Reading	18,300 (7.1%)	2,563 (6.6%)	599 (5.7%)	1,417 (4.2%)	
Bury	13,222 (5.2%)	2,171 (5.6%)	675 (6.5%)	2,293 (6.8%)	
Newcastle	18,594 (7.2%)	3,149 (8.1%)	800 (7.7%)	2,610 (7.8%)	
Leeds	18,795 (7.3%)	2,588 (6.7%)	720 (6.9%)	3,326 (9.9%)	
Bristol	25,933 (10.1%)	3,718 (9.6%)	830 (8.0%)	2,179 (6.5%)	
Barts	7,661 (3.0%)	1,033 (2.7%)	387 (3.7%)	554 (1.7%)	
Nottingham	14,751 (5.7%)	1,895 (4.9%)	566 (5.4%)	2,738 (8.2%)	
Sheffield	13,867 (5.4%)	1,921 (5.0%)	508 (4.9%)	2,310 (6.9%)	
Liverpool	17,191 (6.7%)	3,014 (7.8%)	783 (7.5%)	2,348 (7.0%)	
Middlesborough	8,483 (3.3%)	1,376 (3.5%)	319 (3.1%)	1,822 (5.4%)	
Hounslow	14,922 (5.8%)	1,832 (4.7%)	850 (8.2%)	1,743 (5.2%)	
Croydon	16,210 (6.3%)	2,472 (6.4%)	772 (7.4%)	1,290 (3.9%)	
Birmingham	12,239 (4.8%)	2,131 (5.5%)	724 (6.9%)	1,641 (4.9%)	
Swansea	809 (0.3%)	131 (0.3%)	26 (0.2%)	231 (0.7%)	
Wrexham	254 (0.1%)	41 (0.1%)	7 (0.1%)	46 (0.1%)	
Age at recruitment (years)	54.949 (8.022)	50.861 (5.893)	57.802 (7.468)	63.278 (5.166)	<0.001
Female	147,518 (57.4%)	21,570 (55.6%)	4,155 (40.5%)	13,161 (39.3%)	<0.001
Female before menopause	48,989 (19.1%)	9,768 (25.2%)	823 (8.0%)	652 (1.9%)	<0.001
Waist circumference (cm)	84.469 (10.207)	102.611 (10.216)	91.394 (9.511)	101.778 (14.133)	<0.001
Standing height (cm)	168.597 (9.159)	168.530 (9.435)	169.114 (9.309)	169.682 (9.503)	<0.001
Seated height (cm)	137.005 (7.050)	137.783 (7.221)	136.846 (7.415)	137.650 (7.193)	<0.001
Body mass index (BMI)	25.110 (2.708)	33.509 (3.529)	26.532 (2.426)	31.240 (5.539)	<0.001
Pulse rate (bpm)	68.256 (10.507)	71.985 (10.893)	72.196 (11.762)	69.661 (13.173)	<0.001
Total mortality (all causes)	11,548 (4.5%)	1,305 (3.4%)	1,166 (11.4%)	9,371 (28.0%)	<0.001
1-year survival probability	256,271 (99.9%)	38,720 (99.9%)	10,410 (99.8%)	33,410 (99.7%)	
5-year survival probability	253,760 (98.9%)	38,415 (99.1%)	10,201 (97.9%)	32,336 (95.5%)	
10-year survival probability	249,498 (97.2%)	37,962 (97.9%)	9,795 (93.9%)	29,167 (87.0%)	
15-year survival probability	37,228 (95.1%)	5,953 (96.4%)	1,219 (87.3%)	3,532 (68.5 %)	
Systolic blood pressure (mmHg)	134.951 (18.157)	137.589 (16.366)	140.626 (18.102)	144.650 (19.420)	<0.001
Diastolic blood pressure (mmHg)	80.896 (9.867)	86.387 (9.493)	82.236 (9.939)	83.098 (10.712)	<0.001
Pulse pressure (mmHg)	54.055 (12.745)	51.202 (11.342)	58.390 (13.769)	61.550 (15.580)	<0.001
Whole body fat mass (kg)	20.747 (5.958)	35.917 (8.859)	22.309 (5.767)	31.274 (11.144)	<0.001
Whole body fat-free mass (kg)	50.989 (10.554)	59.394 (12.198)	53.920 (10.417)	58.775 (12.120)	<0.001
Whole body water mass (kg)	37.304 (7.716)	43.493 (8.920)	39.457 (7.617)	43.043 (8.888)	<0.001
Sleep apnoea	487 (0.2%)	224 (0.6%)	75 (0.7%)	568 (1.7%)	<0.001
Chronic ischemic heart disease	1,883 (0.7%)	189 (0.5%)	384 (3.7%)	5,074 (15.2%)	<0.001
Nonrheumatic mitral valve disease	139 (0.1%)	7 (0.0%)	8 (0.1%)	547 (1.6%)	<0.001
Nonrheumatic aortic valve disease	108 (0.0%)	15 (0.0%)	10 (0.1%)	489 (1.5%)	<0.001
Cardiomyopathy	28 (0.0%)	9 (0.0%)	2 (0.0%)	355 (1.1%)	<0.001
Varicose veins of lower extremities	4,618 (1.8%)	666 (1.7%)	165 (1.6%)	840 (2.5%)	<0.001
Hypotension	343 (0.1%)	41 (0.1%)	33 (0.3%)	353 (1.1%)	<0.001
Angina pectoris/ coronary artery disease	1,561 (0.6%)	185 (0.5%)	288 (2.8%)	3,843 (11.5%)	<0.001
Endocrine, nutritional and metabolic diseases	6,579 (2.6%)	1,590 (4.1%)	2,412 (23.5%)	8,116 (24.2%)	<0.001
Mental and behavioural disorders	3,516 (1.4%)	810 (2.1%)	362 (3.5%)	1,461 (4.4%)	<0.001
Diseases of the nervous system	7,745 (3.0%)	1,767 (4.6%)	644 (6.3%)	3,322 (9.9%)	<0.001
Diseases of the eye and adnexa	8,308 (3.2%)	990 (2.6%)	761 (7.4%)	2,848 (8.5%)	<0.001
Diseases of the respiratory system	12,292 (4.8%)	2,486 (6.4%)	866 (8.5%)	5,221 (15.6%)	<0.001
Diseases of the digestive system	43,157 (16.8%)	7,279 (18.8%)	2,779 (27.1%)	11,466 (34.2%)	<0.001
Diseases of the skin and subcutaneous tissue	11,278 (4.4%)	2,041 (5.3%)	680 (6.6%)	3,179 (9.5%)	<0.001
Diseases of the musculoskeletal system and connective tissue	26,477 (10.3%)	4,865 (12.5%)	1,720 (16.8%)	8,800 (26.3%)	<0.001
Diseases of the genitourinary system	35,332 (13.8%)	6,051 (15.6%)	1,686 (16.5%)	6,842 (20.4%)	<0.001
Pregnancy, childbirth and the puerperium	12,029 (4.7%)	1,974 (5.1%)	158 (1.5%)	64 (0.2%)	<0.001
Aortic aneurysm	60 (0.0%)	3 (0.0%)	6 (0.1%)	189 (0.6%)	<0.001
Phlebitis and thrombophlebitis	1,268 (0.5%)	271 (0.7%)	80 (0.8%)	948 (2.8%)	<0.001
Type 2 diabetes	0 (0.0%)	0 (0.0%)	2,628 (25.6%)	3,820 (11.4%)	<0.001
Myocardial infarction	883 (0.3%)	72 (0.2%)	158 (1.5%)	2,555 (7.6%)	<0.001
Chronic kidney disease	940 (0.4%)	103 (0.3%)	117 (1.1%)	2,055 (6.1%)	<0.001
Hypertensive renal disease	73 (0.0%)	7 (0.0%)	21 (0.2%)	305 (0.9%)	<0.001
Endocarditis	370 (0.1%)	42 (0.1%)	14 (0.1%)	294 (0.9%)	<0.001
Essential (primary) hypertension	6,349 (2.5%)	1,102 (2.8%)	1,216 (11.9%)	10,217 (30.5%)	<0.001
Atrial fibrillation and flutter	0 (0.0%)	0 (0.0%)	0 (0.0%)	5,962 (17.8%)	<0.001
Cerebrovascular event/ stroke	1,053 (0.4%)	149 (0.4%)	127 (1.2%)	1,371 (4.1%)	<0.001
Embolism and thrombosis	2,436 (0.9%)	448 (1.2%)	154 (1.5%)	1,691 (5.0%)	<0.001

Pulmonary embolism	504 (0.2%)	111 (0.3%)	30 (0.3%)	466 (1.4%)	<0.001
Pulmonary artery hypertension	22 (0.0%)	4 (0.0%)	2 (0.0%)	123 (0.4%)	<0.001
Neoplasms	33,946 (13.2%)	4,750 (12.3%)	1,591 (15.5%)	6,640 (19.8%)	<0.001
Congenital malformations, deformations and chromosomal abnormalities	1,390 (0.5%)	236 (0.6%)	59 (0.6%)	475 (1.4%)	<0.001
Congenital malformations of the circulatory system	221 (0.1%)	31 (0.1%)	9 (0.1%)	189 (0.6%)	<0.001
Seizure/Epilepsy	1,565 (0.6%)	276 (0.7%)	107 (1.0%)	556 (1.7%)	<0.001
Swollen ankle region	0 (0.0%)	0 (0.0%)	0 (0.0%)	1,212 (3.6%)	<0.001
Impaired exercise tolerance	0 (0.0%)	0 (0.0%)	0 (0.0%)	1,448 (4.3%)	<0.001
Joint swelling	2 (0.0%)	0 (0.0%)	0 (0.0%)	4 (0.0%)	<0.001
Tachycardia	146 (0.1%)	28 (0.1%)	18 (0.2%)	134 (0.4%)	<0.001
Bradycardia	183 (0.1%)	23 (0.1%)	15 (0.1%)	331 (1.0%)	<0.001
Palpitations	515 (0.2%)	92 (0.2%)	36 (0.4%)	585 (1.7%)	<0.001
Cough	351 (0.1%)	73 (0.2%)	24 (0.2%)	241 (0.7%)	<0.001
Symptoms and signs involving the digestive system and abdomen	14,892 (5.8%)	2,587 (6.7%)	895 (8.7%)	3,695 (11.0%)	<0.001
Symptoms and signs involving the skin and subcutaneous tissue	1,418 (0.6%)	292 (0.8%)	107 (1.0%)	516 (1.5%)	<0.001
Symptoms and signs involving the urinary system	6,912 (2.7%)	929 (2.4%)	468 (4.6%)	2,414 (7.2%)	<0.001
Symptoms and signs involving cognition, perception, emotional state and behaviour	964 (0.4%)	184 (0.5%)	85 (0.8%)	631 (1.9%)	<0.001
Symptoms and signs involving speech and voice	406 (0.2%)	70 (0.2%)	35 (0.3%)	233 (0.7%)	<0.001
Dyspnoea	0 (0.0%)	0 (0.0%)	0 (0.0%)	3,537 (10.6%)	<0.001
General, local, unspecified oedema	0 (0.0%)	0 (0.0%)	0 (0.0%)	163 (0.5%)	<0.001
Nocturia	0 (0.0%)	0 (0.0%)	0 (0.0%)	459 (1.4%)	<0.001
Post-viral fatigue	794 (0.3%)	84 (0.2%)	25 (0.2%)	130 (0.4%)	<0.001
Syncope	0 (0.0%)	0 (0.0%)	0 (0.0%)	1,574 (4.7%)	<0.001
Fatigue (excl. post-viral)	18 (0.0%)	5 (0.0%)	2 (0.0%)	2,510 (7.5%)	<0.001
Cholesterol-lowering medication	20,230 (7.9%)	2,865 (7.4%)	4,428 (42.5%)	15,511 (46.3%)	<0.001
Mineralocorticoid receptor antagonist	52 (0.0%)	6 (0.0%)	4 (0.0%)	367 (1.1%)	<0.001
Levothyroxine	2,732 (1.1%)	452 (1.2%)	151 (1.5%)	1,364 (4.1%)	<0.001
Metformin	64 (0.0%)	29 (0.1%)	575 (5.6%)	1,696 (5.1%)	<0.001
Warfarin	308 (0.1%)	66 (0.2%)	12 (0.1%)	1,916 (5.7%)	<0.001
Sulfonylurea	27 (0.0%)	4 (0.0%)	324 (3.2%)	931 (2.8%)	<0.001
Iron therapy	4,985 (1.9%)	659 (1.7%)	185 (1.8%)	1,448 (4.3%)	<0.001
Beta blocker	10,441 (4.1%)	1,111 (2.9%)	832 (8.1%)	12,954 (38.7%)	<0.001
ACE Inhibitor	11,607 (4.5%)	1,688 (4.4%)	2,005 (19.6%)	13,237 (39.5%)	<0.001
Angiotensin receptor blocker (ARB)	3,753 (1.5%)	504 (1.3%)	612 (6.0%)	5,292 (15.8%)	<0.001
Loop diuretic	0 (0.0%)	0 (0.0%)	0 (0.0%)	3,846 (11.5%)	<0.001
Calcium channel blocker	8,825 (3.4%)	876 (2.3%)	926 (9.0%)	10,150 (30.3%)	<0.001
Aspirin	19,790 (7.7%)	2,781 (7.2%)	2,841 (27.7%)	14,238 (42.5%)	<0.001
Thiazide diuretic	6,479 (2.5%)	533 (1.4%)	573 (5.6%)	8,988 (26.8%)	<0.001
Cardiac output	4.631 (1.152)	5.160 (1.670)	4.786 (1.804)	4.779 (1.250)	<0.001
Cardiac index	2.545 (0.551)	2.531 (0.538)	2.541 (0.871)	2.435 (0.587)	<0.001
LV end diastolic volume	144.684 (32.106)	158.659 (32.522)	141.373 (31.585)	159.609 (37.254)	<0.001
LV end systolic volume	58.221 (16.848)	63.977 (17.156)	56.846 (16.338)	66.105 (20.537)	<0.001
LV stroke volume	86.463 (18.577)	94.682 (19.229)	84.527 (18.961)	93.505 (21.346)	<0.001
LV ejection fraction	60.075 (5.273)	59.921 (5.220)	60.066 (5.522)	58.950 (6.102)	<0.001
LV cardiac output	5.320 (1.199)	5.964 (1.322)	5.579 (1.305)	5.736 (1.362)	<0.001
LV myocardial mass	82.540 (20.672)	95.180 (23.160)	88.074 (19.339)	98.125 (24.670)	<0.001
RV end diastolic volume	154.212 (36.767)	168.690 (37.288)	150.630 (34.570)	165.980 (39.006)	<0.001
RV end systolic volume	65.940 (20.595)	72.642 (21.124)	64.483 (18.981)	72.929 (22.540)	<0.001
RV stroke volume	88.272 (19.743)	96.047 (20.331)	86.147 (19.344)	93.050 (22.715)	<0.001
RV ejection fraction	57.700 (5.682)	57.303 (5.602)	57.530 (5.754)	56.381 (7.155)	<0.001
LA maximum volume	69.469 (20.550)	80.686 (22.073)	67.463 (20.330)	92.448 (35.504)	<0.001
LA minimum volume	26.905 (11.686)	31.865 (12.821)	26.184 (11.874)	46.564 (30.488)	<0.001
LA stroke volume	42.564 (11.233)	48.821 (11.974)	41.279 (11.067)	45.488 (13.341)	<0.001
LA ejection fraction	62.361 (8.268)	61.487 (8.109)	62.393 (8.875)	52.826 (13.987)	<0.001
RA maximum volume	86.283 (26.514)	84.043 (25.918)	80.342 (25.984)	97.609 (37.551)	<0.001
RA minimum volume	45.874 (17.578)	44.313 (16.392)	42.314 (16.526)	56.462 (31.661)	<0.001
RA stroke volume	40.409 (13.015)	39.731 (13.564)	38.027 (13.236)	41.147 (14.559)	<0.001
RA ejection fraction	47.418 (8.958)	47.650 (9.287)	47.924 (9.353)	44.243 (11.815)	<0.001
LV mean myocardial wall thickness global	5.554 (0.715)	5.988 (0.797)	5.945 (0.710)	6.164 (0.862)	<0.001
LV circumferential strain AHA 1	-23.293 (5.056)	-23.530 (5.503)	-22.614 (5.548)	-23.071 (5.998)	<0.001
LV circumferential strain AHA 2	-22.044 (6.991)	-20.175 (7.413)	-20.328 (6.857)	-18.888 (7.575)	<0.001
LV circumferential strain AHA 3	-20.005 (5.630)	-19.336 (5.630)	-19.651 (5.442)	-18.323 (5.822)	<0.001
LV circumferential strain global	-22.603 (2.995)	-22.289 (2.940)	-22.321 (3.190)	-21.541 (3.732)	<0.001
LV radial strain global	45.375 (7.722)	45.258 (7.669)	45.502 (8.164)	44.414 (9.202)	<0.001
LV longitudinal strain global	-18.595 (2.590)	-19.015 (2.650)	-18.287 (2.598)	-18.038 (3.084)	<0.001

Supplementary Table 6: Performance evaluation of nested 10-fold cross-validation on the training cohort for different modality combinations

This table summarises the performance metrics of the nested 10-fold cross-validation experiment conducted on the training cohort (n = 401,917) for various combinations of data modalities. The performance metrics include the mean, 95% confidence interval (CI), and p-value from a Mann-Whitney U test. The p-values compare each model's performance to that of the full multi-omics classifier (LAB + PHE + MET + PRO). The following metrics are provided: accuracy, F1 score, negative predictive value (NPV), precision, receiver operating characteristic area under the curve (ROC AUC), sensitivity, and specificity.

Score	Modality	Mean	95% CI Lower	95% CI Upper	p-value (Mann-Whitney U test)
Accuracy	HxQ	0,7314	0,7301	0,7329	0,000183
	LAB	0,7249	0,723	0,7267	0,000183
	LAB + PHE + MET + PRO	0,8502	0,8474	0,8535	
	MET	0,698	0,6963	0,6995	0,000183
	PHE	0,7445	0,7426	0,7463	0,000183
	PRO	0,8344	0,8313	0,8373	0,000182
	RFE	0,8519	0,8483	0,856	0,623176
	SNPs + PRS + TEL	0,6061	0,6043	0,6077	0,000183
Brier Score	HxQ	0,1736	0,1731	0,1741	0,000183
	LAB	0,1843	0,1835	0,185	0,000183
	LAB + PHE + MET + PRO	0,1104	0,1087	0,1119	
	MET	0,2025	0,202	0,2029	0,000183
	PHE	0,1605	0,1594	0,1616	0,000183
	PRO	0,1203	0,119	0,1217	0,000183
	RFE	0,1081	0,1057	0,11	0,185877
	SNPs + PRS + TEL	0,2332	0,2329	0,2334	0,000183
F1 Score	HxQ	0,2739	0,2721	0,2761	0,000183
	LAB	0,2587	0,2567	0,2608	0,000183
	LAB + PHE + MET + PRO	0,3957	0,3885	0,4029	
	MET	0,2273	0,2262	0,2285	0,000183
	PHE	0,293	0,2904	0,2951	0,000183
	PRO	0,3622	0,3571	0,3684	0,000246
	RFE	0,399	0,3912	0,4068	0,623176
	SNPs + PRS + TEL	0,1701	0,1686	0,1715	0,000183
NPV	HxQ	0,9788	0,9783	0,9793	0,000183
	LAB	0,9724	0,9719	0,9729	0,000183
	LAB + PHE + MET + PRO	0,9879	0,9865	0,9892	
	MET	0,967	0,9666	0,9673	0,000183
	PHE	0,983	0,9823	0,9836	0,000246
	PRO	0,9851	0,9843	0,9859	0,01133
	RFE	0,9884	0,9875	0,9892	0,520523
	SNPs + PRS + TEL	0,9552	0,9546	0,9558	0,000183
Precision	HxQ	0,1663	0,1652	0,1676	0,000183
	LAB	0,1579	0,1567	0,1591	0,000183
	LAB + PHE + MET + PRO	0,2595	0,2546	0,2646	
	MET	0,1371	0,1364	0,1378	0,000183
	PHE	0,1786	0,1769	0,1799	0,000183
	PRO	0,2343	0,2305	0,2388	0,000246
	RFE	0,2618	0,2562	0,2679	0,623176
	SNPs + PRS + TEL	0,099	0,0982	0,0998	0,000183
ROC AUC	HxQ	0,8308	0,8282	0,8335	0,000183
	LAB	0,7961	0,7938	0,7982	0,000183
	LAB + PHE + MET + PRO	0,9205	0,915	0,9263	
	MET	0,7479	0,7464	0,7494	0,000183
	PHE	0,8626	0,8601	0,8648	0,000183
	PRO	0,8991	0,8951	0,9034	0,000246
	RFE	0,9236	0,9199	0,9274	0,384673
	SNPs + PRS + TEL	0,6465	0,6427	0,6502	0,000183
Sensitivity	HxQ	0,7744	0,7696	0,7796	0,000246
	LAB	0,7143	0,709	0,7197	0,000183
	LAB + PHE + MET + PRO	0,8328	0,8143	0,8523	
	MET	0,6654	0,6614	0,6692	0,000183
	PHE	0,8157	0,8087	0,8228	0,241322
	PRO	0,7984	0,7871	0,8093	0,023083
	RFE	0,8391	0,8266	0,8497	0,519895
	SNPs + PRS + TEL	0,6035	0,598	0,6091	0,000182
Specificity	HxQ	0,7284	0,727	0,73	0,000181
	LAB	0,7257	0,7237	0,7278	0,000181
	LAB + PHE + MET + PRO	0,8513	0,8479	0,8549	
	MET	0,7003	0,6983	0,7021	0,000181
	PHE	0,7395	0,7375	0,7416	0,000181
	PRO	0,8366	0,8332	0,8399	0,000242
	RFE	0,8527	0,8486	0,857	0,909654
	SNPs + PRS + TEL	0,6063	0,6046	0,6081	0,000181

Supplementary Table 7: Performance evaluation of classifiers validated on the validation cohort for different modality combinations

This table summarises the performance metrics of ten classifiers with random weight initialisation, validated on the validation cohort (n = 7,394) for various combinations of data modalities. The performance metrics include the mean, 95% confidence interval (CI), and p-value from a Mann-Whitney U test. The p-values compare each model's performance to that of the full multi-omics classifier (LAB + PHE + MET + PRO). The following metrics are provided: accuracy, F1 score, negative predictive value (NPV), precision, receiver operating characteristic area under the curve (ROC AUC), sensitivity, and specificity.

Score	Modality	Mean	95% CI Lower	95% CI Upper	p-value (Mann-Whitney U test)
Accuracy	HxQ	0,7034	0,7028	0,7042	0,000174
	LAB	0,7286	0,7277	0,7295	0,000179
	LAB + PHE + MET + PRO	0,8471	0,8463	0,848	
	MET	0,6769	0,6761	0,6777	0,000178
	PHE	0,7239	0,7232	0,7246	0,000178
	PRO	0,8371	0,8359	0,8383	0,000179
	RFE	0,8522	0,8514	0,8529	0,000179
	SNPs + PRS + TEL	0,6031	0,6022	0,604	0,000177
Brier Score	HxQ	0,188	0,1879	0,1882	0,000183
	LAB	0,1819	0,1817	0,1821	0,000183
	LAB + PHE + MET + PRO	0,1101	0,11	0,1103	
	MET	0,2103	0,2102	0,2104	0,000183
	PHE	0,1708	0,1706	0,1709	0,000183
	PRO	0,1187	0,1184	0,119	0,000183
	RFE	0,1069	0,1066	0,1073	0,000183
	SNPs + PRS + TEL	0,2333	0,2332	0,2334	0,000183
F1 Score	HxQ	0,2429	0,2421	0,2437	0,000178
	LAB	0,241	0,24	0,2417	0,000182
	LAB + PHE + MET + PRO	0,4024	0,4005	0,4042	
	MET	0,2048	0,2038	0,2057	0,000182
	PHE	0,2561	0,2554	0,2567	0,000182
	PRO	0,3763	0,3743	0,3784	0,000182
	RFE	0,4109	0,4097	0,4121	0,000245
	SNPs + PRS + TEL	0,1561	0,1552	0,157	0,000182
NPV	HxQ	0,9813	0,9811	0,9815	0,000178
	LAB	0,9758	0,9756	0,9761	0,000182
	LAB + PHE + MET + PRO	0,9893	0,9891	0,9896	
	MET	0,9718	0,9715	0,972	0,000182
	PHE	0,9818	0,9817	0,9819	0,000182
	PRO	0,9863	0,9861	0,9866	0,000182
	RFE	0,9895	0,9893	0,9897	0,344523
	SNPs + PRS + TEL	0,9604	0,9601	0,9608	0,000182
Precision	HxQ	0,1435	0,143	0,1439	0,000178
	LAB	0,1448	0,1442	0,1453	0,000182
	LAB + PHE + MET + PRO	0,2629	0,2616	0,2644	
	MET	0,1202	0,1196	0,1207	0,000182
	PHE	0,1527	0,1523	0,1532	0,000182
	PRO	0,2444	0,2428	0,246	0,000182
	RFE	0,2701	0,2691	0,2711	0,000182
	SNPs + PRS + TEL	0,0895	0,089	0,09	0,000182
ROC AUC	HxQ	0,8292	0,829	0,8293	0,000183
	LAB	0,7955	0,7949	0,796	0,000183
	LAB + PHE + MET + PRO	0,9308	0,9303	0,9313	
	MET	0,7541	0,7535	0,7546	0,000183
	PHE	0,8441	0,8439	0,8442	0,000183
	PRO	0,9114	0,9107	0,9121	0,000183
	RFE	0,9322	0,9318	0,9325	0,001315
	SNPs + PRS + TEL	0,6448	0,6441	0,6453	0,000183
Sensitivity	HxQ	0,7923	0,7899	0,7948	0,000157
	LAB	0,7173	0,7151	0,72	0,000171
	LAB + PHE + MET + PRO	0,8572	0,8545	0,8601	
	MET	0,6928	0,6899	0,6957	0,000171
	PHE	0,7912	0,7899	0,7928	0,000167
	PRO	0,8182	0,8151	0,8212	0,000171
	RFE	0,8586	0,8559	0,861	0,69981
	SNPs + PRS + TEL	0,6115	0,6079	0,6151	0,000171
Specificity	HxQ	0,6978	0,697	0,6985	0,000177
	LAB	0,7293	0,7283	0,7304	0,00018
	LAB + PHE + MET + PRO	0,8465	0,8457	0,8473	
	MET	0,6759	0,675	0,6767	0,000181
	PHE	0,7196	0,7189	0,7204	0,000181
	PRO	0,8383	0,8372	0,8396	0,000182
	RFE	0,8518	0,851	0,8526	0,000182
	SNPs + PRS + TEL	0,6025	0,6016	0,6035	0,000181

Supplementary Table 8: Extended characteristics of the HFpEF clusters and control groups

Comparison of baseline characteristics between HFpEF Cluster 1 (n = 268), HFpEF Cluster 2 (n = 204), HFpEF Cluster 3 (n = 309), HFpEF Cluster 4 (n = 346), HFpEF Cluster 5 (n = 363), HFpEF Cluster 6 (n = 156), obese controls (n = 38,772), diabetic controls (n = 10,247), and non-HF controls (n = 256,895). The characteristics assessed include age, sex, BMI, mortality rate, symptoms, diagnoses, and medication intake. Continuous variables are presented as mean with standard deviation in the brackets, while binary variables are shown as the number of true cases with the group proportion in the brackets. Differences in continuous variables between groups were analysed using either linear regression main effects testing or the Kruskal–Wallis rank test. Categorical variables were compared using Pearson's χ^2 test or Fisher's exact test, as appropriate.

	HFpEF Cluster 1 (n = 268)	HFpEF Cluster 2 (n = 204)	HFpEF Cluster 3 (n = 309)	HFpEF Cluster 4 (n = 346)	HFpEF Cluster 5 (n = 363)	HFpEF Cluster 6 (n = 156)	Obese controls (n = 38,772)	Diabetic controls (n = 10,247)	Non-HF controls (n = 256,895)	p-value
Age at recruitment (years)	63.716 (4.996)	63.873 (4.801)	62.291 (5.331)	64.272 (4.329)	64.835 (4.453)	64.654 (4.666)	50.861 (5.893)	57.802 (7.468)	54.949 (8.022)	<0.001
Female	87 (32.5%)	93 (45.6%)	6 (1.9%)	328 (94.8%)	16 (4.4%)	144 (92.3%)	21,570 (55.6%)	4,155 (40.5%)	147,518 (57.4%)	<0.001
Female before menopause	3 (1.1%)	6 (2.9%)	0 (0.0%)	14 (4.0%)	3 (0.8%)	7 (4.5%)	9,768 (25.2%)	823 (8.0%)	48,989 (19.1%)	<0.001
Waist circumference (cm)	101.722 (11.363)	109.805 (15.935)	113.569 (10.056)	101.997 (10.696)	99.223 (10.064)	79.157 (9.063)	102.611 (10.216)	91.394 (9.511)	84.469 (10.207)	<0.001
Standing height (cm)	170.239 (8.031)	167.025 (10.249)	177.285 (6.377)	160.848 (6.107)	174.066 (6.686)	162.631 (6.754)	168.530 (9.435)	169.114 (9.309)	168.597 (9.159)	<0.001
Seated height (cm)	138.022 (6.590)	135.482 (8.060)	142.656 (4.545)	131.736 (6.628)	140.131 (5.492)	133.160 (6.426)	137.783 (7.221)	136.846 (7.415)	137.005 (7.050)	<0.001
Body mass index (BMI)	30.672 (4.152)	34.877 (6.847)	34.317 (3.961)	34.250 (4.551)	28.582 (3.753)	24.590 (2.990)	33.509 (3.529)	26.532 (2.426)	25.110 (2.708)	<0.001
Pulse rate (bpm)	69.724 (13.375)	73.983 (13.951)	70.951 (12.780)	70.122 (13.587)	65.066 (12.915)	64.016 (9.547)	71.985 (10.893)	72.196 (11.762)	68.256 (10.507)	<0.001
Systolic blood pressure (mmHg)	147.858 (20.282)	140.172 (20.971)	146.945 (18.237)	147.736 (20.723)	141.105 (20.350)	139.333 (20.633)	137.589 (16.366)	140.626 (18.102)	134.951 (18.157)	<0.001
Diastolic blood pressure (mmHg)	84.058 (11.700)	77.948 (12.182)	87.283 (9.653)	84.204 (9.840)	81.324 (11.484)	76.609 (9.986)	86.387 (9.493)	82.236 (9.939)	80.896 (9.867)	<0.001
Pulse pressure (mmHg)	63.800 (16.279)	62.224 (16.956)	59.662 (14.576)	63.532 (17.909)	59.781 (15.527)	62.724 (15.812)	51.202 (11.342)	58.390 (13.769)	54.055 (12.745)	<0.001
Whole body fat mass (kg)	30.051 (8.889)	37.266 (13.026)	35.588 (8.356)	39.692 (9.178)	23.688 (7.161)	21.542 (6.150)	35.917 (8.859)	22.309 (5.767)	20.747 (5.958)	<0.001
Whole body fat-free mass (kg)	59.356 (10.390)	59.627 (12.926)	72.507 (7.473)	48.871 (5.267)	62.624 (7.144)	43.428 (5.251)	59.394 (12.198)	53.920 (10.417)	50.989 (10.554)	<0.001
Whole body water mass (kg)	43.443 (7.571)	43.828 (9.679)	53.100 (5.516)	35.832 (3.901)	45.840 (5.237)	31.779 (3.850)	43.493 (8.920)	39.457 (7.617)	37.304 (7.716)	<0.001
Death (all causes)	80 (29.9%)	133 (65.2%)	85 (27.5%)	83 (24.0%)	110 (30.3%)	23 (14.7%)	1,305 (3.4%)	1,166 (11.4%)	11,548 (4.5%)	<0.001
Sleep apnoea	2 (0.7%)	10 (4.9%)	9 (2.9%)	3 (0.9%)	2 (0.6%)	0 (0.0%)	224 (0.6%)	75 (0.7%)	487 (0.2%)	<0.001
Chronic ischemic heart disease	46 (17.2%)	51 (25.0%)	54 (17.5%)	36 (10.4%)	95 (26.2%)	11 (7.1%)	189 (0.5%)	384 (3.7%)	1,883 (0.7%)	<0.001
Nonrheumatic mitral valve disease	6 (2.2%)	6 (2.9%)	6 (1.9%)	11 (3.2%)	20 (5.5%)	10 (6.4%)	7 (0.0%)	8 (0.1%)	139 (0.1%)	<0.001
Nonrheumatic aortic valve disease	3 (1.1%)	3 (1.5%)	3 (1.0%)	5 (1.4%)	6 (1.7%)	0 (0.0%)	15 (0.0%)	10 (0.1%)	108 (0.0%)	<0.001
Cardiomyopathy	1 (0.4%)	6 (2.9%)	10 (3.2%)	4 (1.2%)	6 (1.7%)	2 (1.3%)	9 (0.0%)	2 (0.0%)	28 (0.0%)	<0.001
Varicose veins of lower extremities	4 (1.5%)	4 (2.0%)	6 (1.9%)	13 (3.8%)	10 (2.8%)	5 (3.2%)	666 (1.7%)	165 (1.6%)	4,618 (1.8%)	0.066
Hypotension	1 (0.4%)	3 (1.5%)	2 (0.6%)	4 (1.2%)	5 (1.4%)	3 (1.9%)	41 (0.1%)	33 (0.3%)	343 (0.1%)	<0.001
Angina pectoris/ coronary artery disease	36 (13.4%)	50 (24.5%)	35 (11.3%)	42 (12.1%)	62 (17.1%)	11 (7.1%)	185 (0.5%)	288 (2.8%)	1,561 (0.6%)	<0.001
Endocrine, nutritional and metabolic diseases	62 (23.1%)	111 (54.4%)	79 (25.6%)	79 (22.8%)	98 (27.0%)	26 (16.7%)	1,590 (4.1%)	2,412 (23.5%)	6,579 (2.6%)	<0.001
Mental and behavioural disorders	18 (6.7%)	19 (9.3%)	14 (4.5%)	8 (2.3%)	14 (3.9%)	9 (5.8%)	810 (2.1%)	362 (3.5%)	3,516 (1.4%)	<0.001
Diseases of the nervous system	21 (7.8%)	39 (19.1%)	32 (10.4%)	37 (10.7%)	32 (8.8%)	9 (5.8%)	1,767 (4.6%)	644 (6.3%)	7,745 (3.0%)	<0.001
Diseases of the eye and adnexa	20 (7.5%)	37 (18.1%)	23 (7.4%)	31 (9.0%)	26 (7.2%)	14 (9.0%)	990 (2.6%)	761 (7.4%)	8,308 (3.2%)	<0.001
Diseases of the respiratory system	40 (14.9%)	64 (31.4%)	42 (13.6%)	53 (15.3%)	68 (18.7%)	29 (18.6%)	2,486 (6.4%)	866 (8.5%)	12,292 (4.8%)	<0.001
Diseases of the digestive system	90 (33.6%)	94 (46.1%)	98 (31.7%)	128 (37.0%)	120 (33.1%)	42 (26.9%)	7,279 (18.8%)	2,779 (27.1%)	43,157 (16.8%)	<0.001
Diseases of the skin and subcutaneous tissue	25 (9.3%)	39 (19.1%)	36 (11.7%)	34 (9.8%)	34 (9.4%)	5 (3.2%)	2,041 (5.3%)	680 (6.6%)	11,278 (4.4%)	<0.001
Diseases of the musculoskeletal system and connective tissue	79 (29.5%)	80 (39.2%)	89 (28.8%)	107 (30.9%)	84 (23.1%)	36 (23.1%)	4,865 (12.5%)	1,720 (16.8%)	26,477 (10.3%)	<0.001
Diseases of the genitourinary system	65 (24.3%)	65 (31.9%)	42 (13.6%)	95 (27.5%)	58 (16.0%)	37 (23.7%)	6,051 (15.6%)	1,686 (16.5%)	35,332 (13.8%)	<0.001
Pregnancy, childbirth and the puerperium	0 (0.0%)	0 (0.0%)	0 (0.0%)	1 (0.3%)	0 (0.0%)	0 (0.0%)	1,974 (5.1%)	158 (1.5%)	12,029 (4.7%)	<0.001
Congenital malformations, deformations and chromosomal abnormalities	3 (1.1%)	6 (2.9%)	1 (0.3%)	9 (2.6%)	6 (1.7%)	5 (3.2%)	236 (0.6%)	59 (0.6%)	1,390 (0.5%)	<0.001
Aortic aneurysm	4 (1.5%)	2 (1.0%)	2 (0.6%)	0 (0.0%)	1 (0.3%)	0 (0.0%)	3 (0.0%)	6 (0.1%)	60 (0.0%)	<0.001
Phlebitis and thrombophlebitis	10 (3.7%)	14 (6.9%)	8 (2.6%)	10 (2.9%)	10 (2.8%)	3 (1.9%)	271 (0.7%)	80 (0.8%)	1,268 (0.5%)	<0.001
Type 2 diabetes	31 (11.6%)	87 (42.6%)	45 (14.6%)	22 (6.4%)	28 (7.7%)	3 (1.9%)	0 (0.0%)	2,628 (25.6%)	0 (0.0%)	<0.001
Myocardial infarction	17 (6.3%)	26 (12.7%)	34 (11.0%)	19 (5.5%)	67 (18.5%)	4 (2.6%)	72 (0.2%)	158 (1.5%)	883 (0.3%)	<0.001
Chronic kidney disease	15 (5.6%)	48 (23.5%)	7 (2.3%)	20 (5.8%)	20 (5.5%)	11 (7.1%)	103 (0.3%)	117 (1.1%)	940 (0.4%)	<0.001
Hypertensive renal disease	2 (0.7%)	15 (7.4%)	2 (0.6%)	1 (0.3%)	2 (0.6%)	1 (0.6%)	7 (0.0%)	21 (0.2%)	73 (0.0%)	<0.001
Endocarditis	4 (1.5%)	2 (1.0%)	4 (1.3%)	2 (0.6%)	6 (1.7%)	6 (3.8%)	42 (0.1%)	14 (0.1%)	370 (0.1%)	<0.001
Essential (primary) hypertension	96 (35.8%)	114 (55.9%)	103 (33.3%)	113 (32.7%)	122 (33.6%)	38 (24.4%)	1,102 (2.8%)	1,216 (11.9%)	6,349 (2.5%)	<0.001
Atrial fibrillation and flutter	63 (23.5%)	69 (33.8%)	80 (25.9%)	53 (15.3%)	132 (36.4%)	48 (30.8%)	0 (0.0%)	0 (0.0%)	0 (0.0%)	<0.001
Cerebrovascular event/ stroke	15 (5.6%)	17 (8.3%)	13 (4.2%)	8 (2.3%)	26 (7.2%)	5 (3.2%)	149 (0.4%)	127 (1.2%)	1,053 (0.4%)	<0.001
Embolism and thrombosis	18 (6.7%)	24 (11.8%)	15 (4.9%)	21 (6.1%)	22 (6.1%)	8 (5.1%)	448 (1.2%)	154 (1.5%)	2,436 (0.9%)	<0.001
Pulmonary embolism	4 (1.5%)	8 (3.9%)	2 (0.6%)	7 (2.0%)	3 (0.8%)	4 (2.6%)	111 (0.3%)	30 (0.3%)	504 (0.2%)	<0.001
Pulmonary artery hypertension	3 (1.1%)	12 (5.9%)	5 (1.6%)	11 (3.2%)	9 (2.5%)	8 (5.1%)	4 (0.0%)	2 (0.0%)	22 (0.0%)	<0.001
Neoplasms	50 (18.7%)	49 (24.0%)	48 (15.5%)	73 (21.1%)	62 (17.1%)	36 (23.1%)	4,750 (12.3%)	1,591 (15.5%)	33,946 (13.2%)	<0.001
Congenital malformations of the circulatory system	2 (0.7%)	3 (1.5%)	0 (0.0%)	8 (2.3%)	3 (0.8%)	4 (2.6%)	31 (0.1%)	9 (0.1%)	221 (0.1%)	<0.001
Seizure/Epilepsy	4 (1.5%)	3 (1.5%)	7 (2.3%)	3 (0.9%)	8 (2.2%)	2 (1.3%)	276 (0.7%)	107 (1.0%)	1,565 (0.6%)	<0.001
Swollen ankle region	9 (3.4%)	10 (4.9%)	9 (2.9%)	18 (5.2%)	8 (2.2%)	3 (1.9%)	0 (0.0%)	0 (0.0%)	0 (0.0%)	<0.001
Impaired exercise tolerance	8 (3.0%)	7 (3.4%)	11 (3.6%)	20 (5.8%)	14 (3.9%)	2 (1.3%)	0 (0.0%)	0 (0.0%)	0 (0.0%)	<0.001
Joint swelling	0 (0.0%)	0 (0.0%)	0 (0.0%)	0 (0.0%)	0 (0.0%)	0 (0.0%)	0 (0.0%)	0 (0.0%)	2 (0.0%)	1.000

Tachycardia	1 (0.4%)	1 (0.5%)	2 (0.6%)	2 (0.6%)	2 (0.6%)	0 (0.0%)	28 (0.1%)	18 (0.2%)	146 (0.1%)	<0.001
Bradycardia	2 (0.7%)	8 (3.9%)	3 (1.0%)	2 (0.6%)	5 (1.4%)	3 (1.9%)	23 (0.1%)	15 (0.1%)	183 (0.1%)	<0.001
Palpitations	4 (1.5%)	6 (2.9%)	4 (1.3%)	14 (4.0%)	7 (1.9%)	4 (2.6%)	92 (0.2%)	36 (0.4%)	515 (0.2%)	<0.001
Cough	3 (1.1%)	4 (2.0%)	1 (0.3%)	6 (1.7%)	4 (1.1%)	2 (1.3%)	73 (0.2%)	24 (0.2%)	351 (0.1%)	<0.001
Symptoms and signs involving the digestive system and abdomen	32 (11.9%)	38 (18.6%)	22 (7.1%)	48 (13.9%)	34 (9.4%)	23 (14.7%)	2,587 (6.7%)	895 (8.7%)	14,892 (5.8%)	<0.001
Symptoms and signs involving the skin and subcutaneous tissue	7 (2.6%)	5 (2.5%)	4 (1.3%)	6 (1.7%)	4 (1.1%)	0 (0.0%)	292 (0.8%)	107 (1.0%)	1,418 (0.6%)	<0.001
Symptoms and signs involving the urinary system	21 (7.8%)	22 (10.8%)	20 (6.5%)	24 (6.9%)	41 (11.3%)	7 (4.5%)	929 (2.4%)	468 (4.6%)	6,912 (2.7%)	<0.001
Symptoms and signs involving cognition, perception, emotional state and behaviour	9 (3.4%)	10 (4.9%)	5 (1.6%)	9 (2.6%)	4 (1.1%)	7 (4.5%)	184 (0.5%)	85 (0.8%)	964 (0.4%)	<0.001
Symptoms and signs involving speech and voice	3 (1.1%)	3 (1.5%)	2 (0.6%)	3 (0.9%)	2 (0.6%)	2 (1.3%)	70 (0.2%)	35 (0.3%)	406 (0.2%)	<0.001
Dyspnoea	28 (10.4%)	48 (23.5%)	35 (11.3%)	46 (13.3%)	44 (12.1%)	18 (11.5%)	0 (0.0%)	0 (0.0%)	0 (0.0%)	<0.001
General, local, unspecified oedema	1 (0.4%)	10 (4.9%)	2 (0.6%)	2 (0.6%)	4 (1.1%)	0 (0.0%)	0 (0.0%)	0 (0.0%)	0 (0.0%)	<0.001
Nocturia	1 (0.4%)	3 (1.5%)	3 (1.0%)	2 (0.6%)	10 (2.8%)	1 (0.6%)	0 (0.0%)	0 (0.0%)	0 (0.0%)	<0.001
Post-viral fatigue	0 (0.0%)	0 (0.0%)	2 (0.6%)	1 (0.3%)	2 (0.6%)	1 (0.6%)	84 (0.2%)	25 (0.2%)	794 (0.3%)	0.062
Syncope	17 (6.3%)	12 (5.9%)	14 (4.5%)	13 (3.8%)	17 (4.7%)	11 (7.1%)	0 (0.0%)	0 (0.0%)	0 (0.0%)	<0.001
Fatigue (excl. post-viral)	22 (8.2%)	21 (10.3%)	15 (4.9%)	30 (8.7%)	20 (5.5%)	15 (9.6%)	5 (0.0%)	2 (0.0%)	18 (0.0%)	<0.001
Cholesterol-lowering medication	130 (48.5%)	142 (69.6%)	164 (53.1%)	143 (41.3%)	211 (58.1%)	42 (26.9%)	2,865 (7.4%)	4,342 (42.4%)	20,316 (7.9%)	<0.001
Mineralocorticoid receptor antagonist	5 (1.9%)	18 (8.8%)	4 (1.3%)	1 (0.3%)	7 (1.9%)	2 (1.3%)	6 (0.0%)	4 (0.0%)	52 (0.0%)	<0.001
Levothyroxine	9 (3.4%)	7 (3.4%)	4 (1.3%)	19 (5.5%)	9 (2.5%)	7 (4.5%)	452 (1.2%)	151 (1.5%)	2,732 (1.1%)	<0.001
Metformin	14 (5.2%)	35 (17.2%)	18 (5.8%)	7 (2.0%)	12 (3.3%)	0 (0.0%)	29 (0.1%)	575 (5.6%)	64 (0.0%)	<0.001
Warfarin	18 (6.7%)	28 (13.7%)	26 (8.4%)	19 (5.5%)	52 (14.3%)	12 (7.7%)	66 (0.2%)	12 (0.1%)	308 (0.1%)	<0.001
Sulfonylurea	5 (1.9%)	22 (10.8%)	5 (1.6%)	3 (0.9%)	7 (1.9%)	0 (0.0%)	4 (0.0%)	324 (3.2%)	27 (0.0%)	<0.001
Iron therapy	11 (4.1%)	27 (13.2%)	5 (1.6%)	22 (6.4%)	14 (3.9%)	6 (3.8%)	659 (1.7%)	185 (1.8%)	4,985 (1.9%)	<0.001
Beta blocker	113 (42.2%)	98 (48.0%)	129 (41.7%)	117 (33.8%)	180 (49.6%)	50 (32.1%)	1,111 (2.9%)	832 (8.1%)	10,441 (4.1%)	<0.001
ACE inhibitor	117 (43.7%)	119 (58.3%)	141 (45.6%)	123 (35.5%)	152 (41.9%)	41 (26.3%)	1,688 (4.4%)	2,005 (19.6%)	11,607 (4.5%)	<0.001
Angiotensin receptor blocker (ARB)	37 (13.8%)	51 (25.0%)	49 (15.9%)	49 (14.2%)	64 (17.6%)	16 (10.3%)	504 (1.3%)	612 (6.0%)	3,753 (1.5%)	<0.001
Loop diuretic	26 (9.7%)	75 (36.8%)	40 (12.9%)	52 (15.0%)	55 (15.2%)	8 (5.1%)	0 (0.0%)	0 (0.0%)	0 (0.0%)	<0.001
Calcium channel blocker	97 (36.2%)	79 (38.7%)	89 (28.8%)	81 (23.4%)	105 (28.9%)	28 (17.9%)	876 (2.3%)	926 (9.0%)	8,825 (3.4%)	<0.001
Aspirin	119 (44.4%)	115 (56.4%)	124 (40.1%)	128 (37.0%)	191 (52.6%)	62 (39.7%)	2,781 (7.2%)	2,841 (27.7%)	19,790 (7.7%)	<0.001
Thiazide diuretic	70 (26.1%)	55 (27.0%)	63 (20.4%)	100 (28.9%)	66 (18.2%)	29 (18.6%)	533 (1.4%)	573 (5.6%)	6,479 (2.5%)	<0.001

Supplementary Table 9: Characteristics and top features of HFpEF clusters

Comparison of HFpEF clusters based on basic demographic and health indicators, alongside the performance metrics (ROC AUC, sensitivity, specificity) of two classifiers trained to distinguish cluster cases from non-cluster cases. The first classifier was trained using only non-omics features, with cutoff values derived from dependence plots. The top features selected were those consistently ranking among the top 15 highest mean SHAP values across all five cross-validation folds. The second classifier incorporated both omics and non-omics features, with top features selected based on their consistent ranking in the top 50 highest mean SHAP values. The signs in brackets indicate whether the upregulation (+) or downregulation (-) of an omics feature was predictive of a given cluster.

Cluster		1 (n = 268)	2 (n = 204)	3 (n = 309)	4 (n = 346)	5 (n = 363)	6 (n = 156)
**Basic demographic and health indicators**	Age at recruitment [year] - mean (SD)	63.7 (5.0)	63.9 (4.8)	62.3 (5.3)	64.3 (4.3)	64.8 (4.5)	64.7 (4.7)
	BMI - mean (SD)	30.7 (4.2)	34.9 (6.8)	34.3 (4.0)	34.3 (4.6)	28.6 (3.8)	24.6 (3.0)
	Males [%]	67.5	54.4	98.1	5.2	95.6	7.7
	Mortality rate [%]	29.9	65.2	27.5	24	30.3	14.7
**Catboost performance (non-omics features)**	ROC AUC - mean (95% CI)	0.792 (0.758-0.821)	0.962 (0.957-0.966)	0.899 (0.895-0.906)	0.916 (0.908-0.927)	0.787 (0.754-0.807)	0.768 (0.747-0.793)
	Sensitivity - mean (95% CI)	0.677 (0.609-0.745)	0.855 (0.823-0.892)	0.864 (0.842-0.887)	0.873 (0.859-0.888)	0.645 (0.591-0.680)	0.604 (0.546-0.674)
	Specificity - mean (95% CI)	0.779 (0.770-0.787)	0.926 (0.920-0.931)	0.809 (0.799-0.820)	0.826 (0.817-0.837)	0.779 (0.772-0.787)	0.768 (0.746-0.786)
**Top non-omics features according to mean SHAP (cutoff values)**		Age > 60; Atrial fibrillation and flutter; BMI > 30; Beta blocker; Number of self-reported non-cancer illnesses > 0; Taking other prescription medications	Age > 61; Atrial fibrillation and flutter; BMI > 30; Chronic kidney disease, Other serious medical condition/disability diagnosed by doctor; Overall health rating = Poor/Fair; Taking other prescription medications; Type 2 diabetes; Usual walking pace = Slow	Age > 58; Atrial fibrillation and flutter; BMI > 30, Number of self-reported non-cancer illnesses > 1; Comparative height size at age 10 = Taller; Standard PRS for atrial fibrillation (AF) > 0.5	Age > 60; Atrial fibrillation and flutter; BMI > 30	Age > 60, Atrial fibrillation and flutter; BMI = [20-34]; Beta blocker; Domestic water CaCO3 concentration < 100 mg/L; Other serious medical condition/disability diagnosed by doctor; Standard PRS for atrial fibrillation (AF) > 0.6; Warfarin	Age > 58; Atrial fibrillation and flutter; BMI < 28; Beta blocker; Aspirin; Cereal intake < 11 bowls/week; Standard PRS for atrial fibrillation (AF) > 0.6; Traffic intensity on the nearest major road < 15,000 vehicles/day
**Catboost performance (omics + non-omics features)**	ROC AUC - mean (95% CI)	0.920 (0.908-0.932)	0.988 (0.985-0.990)	0.946 (0.939-0.953)	0.929 (0.920-0.936)	0.866 (0.856-0.874)	0.825 (0.809-0.841)
	Sensitivity - mean (95% CI)	0.821 (0.761-0.896)	0.882 (0.788-0.941)	0.858 (0.803-0.913)	0.862 (0.843-0.878)	0.708 (0.661-0.751)	0.688 (0.638-0.738)
	Specificity - mean (95% CI)	0.839 (0.823-0.853)	0.971 (0.965-0.977)	0.888 (0.877-0.899)	0.851 (0.837-0.863)	0.827 (0.814-0.838)	0.773 (0.739-0.813)
**Top upregulated (+) and downregulated (-) omics and non-omics features according to mean SHAP**		BMI (+), Age (+), HARS1 (+), STAMBP (+)	UMOD (-), EFNA4 (+), TNFRSF10B (+), GDF15 (+), BMI (+), Cystatin C (+), TNFRSF1A (+), FABP4 (+), ANGPT2 (+), ITGBL1 (+)	BMI (+), Weight (+), Age(+), Omega-6 Fatty Acids to Total Fatty Acids percentage (-), Waist circumference (+), NPPB (+), IGFBPL1 (+), KIT (-), PON3 (-), RGS10 (-), TNFAIP8L2 (-)	BMI (+), Weight (+), Age (+), NPPB (+), Waist circumference (+), C-reactive protein (+), Whole body fat mass (+), EDN1 (+), Hip circumference (+)	NPPB (+), Total Fatty Acids (-), Age (+), Aspirin (+), ERBB3 (-), KDR (-), Direct bilirubin (+), Omega-3 Fatty Acids (-)	NPPB (+), BMI (-), Weight (-), Age (+), CC2D1A (-), Monounsaturated Fatty Acids to Total Fatty Acids percentage (-), CKB (+), ITGAV (+), LEPR (+), GLB1 (-), HHEX (-), IGFBP2 (+), NAGK (-), S100A4 (-), SMAD1 (-), Waist circumference (-)

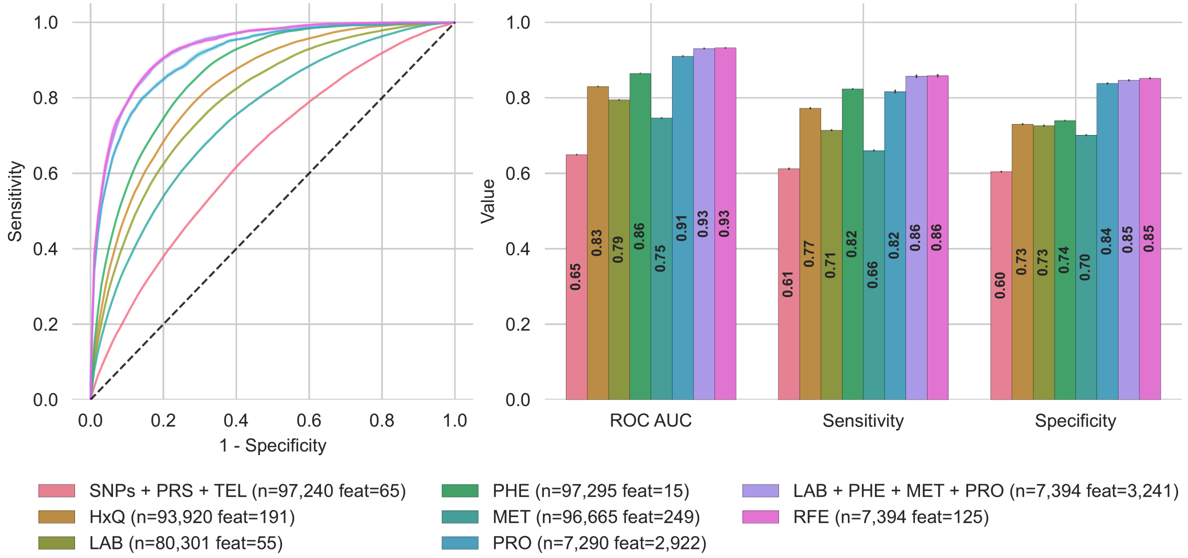

Supplementary Figure 1: Performance metrics on the modality-specific validation cohorts

The classifier was trained ten times on the training cohort using different random weight initializations, and evaluated on the validation cohort. In contrast to Figure 2B, where the validation cohort size was fixed across modalities, here the size of the validation cohort varies by modality depending on data availability.

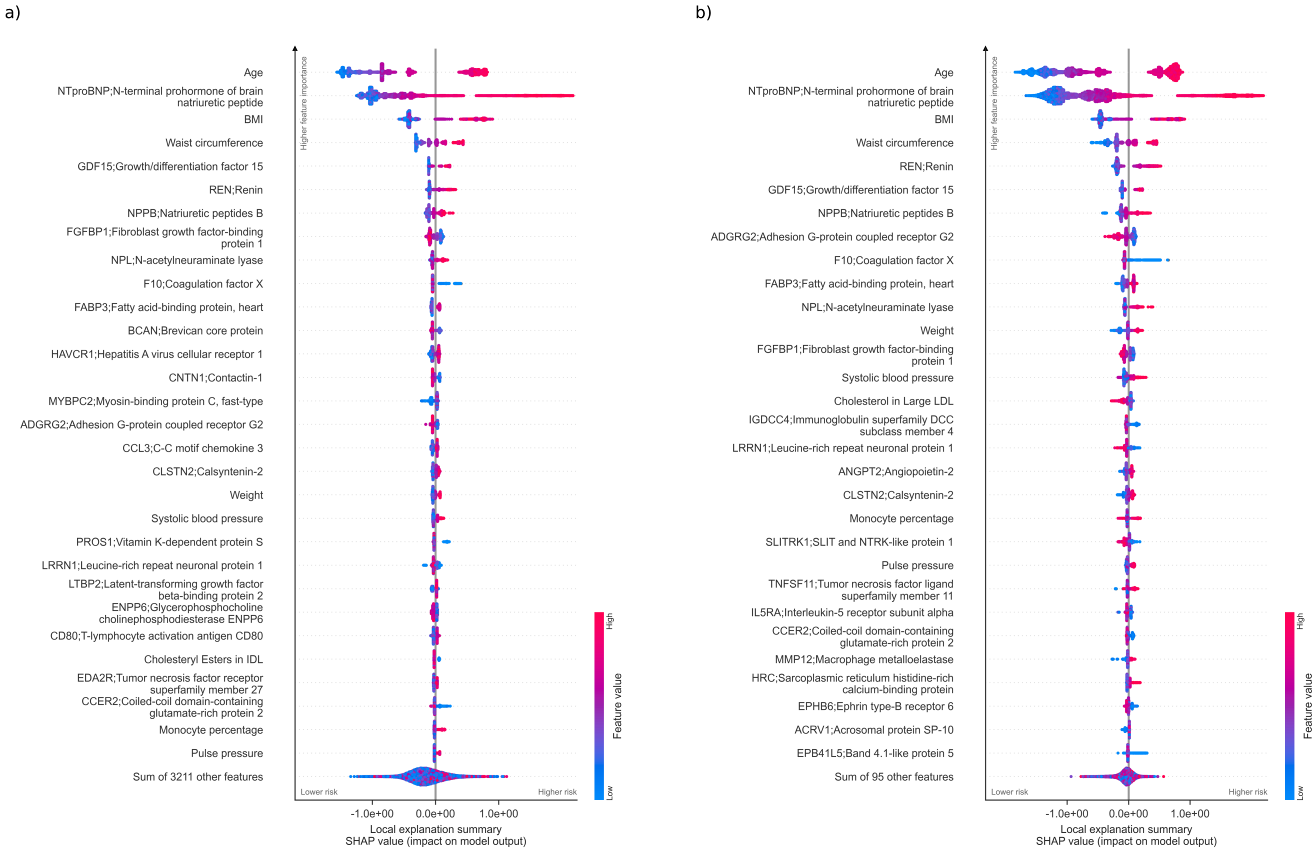

Supplementary Figure 2: SHAP analysis of full and reduced multi-omics classifiers

Comparison of the top 30 multi-omics features with the highest mean SHAP values from the (a) full multi-omics Catboost classifier (3241 omics features) and the (b) RFE classifier (125 omics features). In the scatter plots, red dots represent cases with high feature values, while blue dots represent cases with low feature values. Points leaning to the right indicate that the feature values (high or low, as indicated by colour) contribute positively to the classifier's output, increasing the predicted probability of HFpEF. Points leaning to the left imply a negative contribution to the classifier's output.

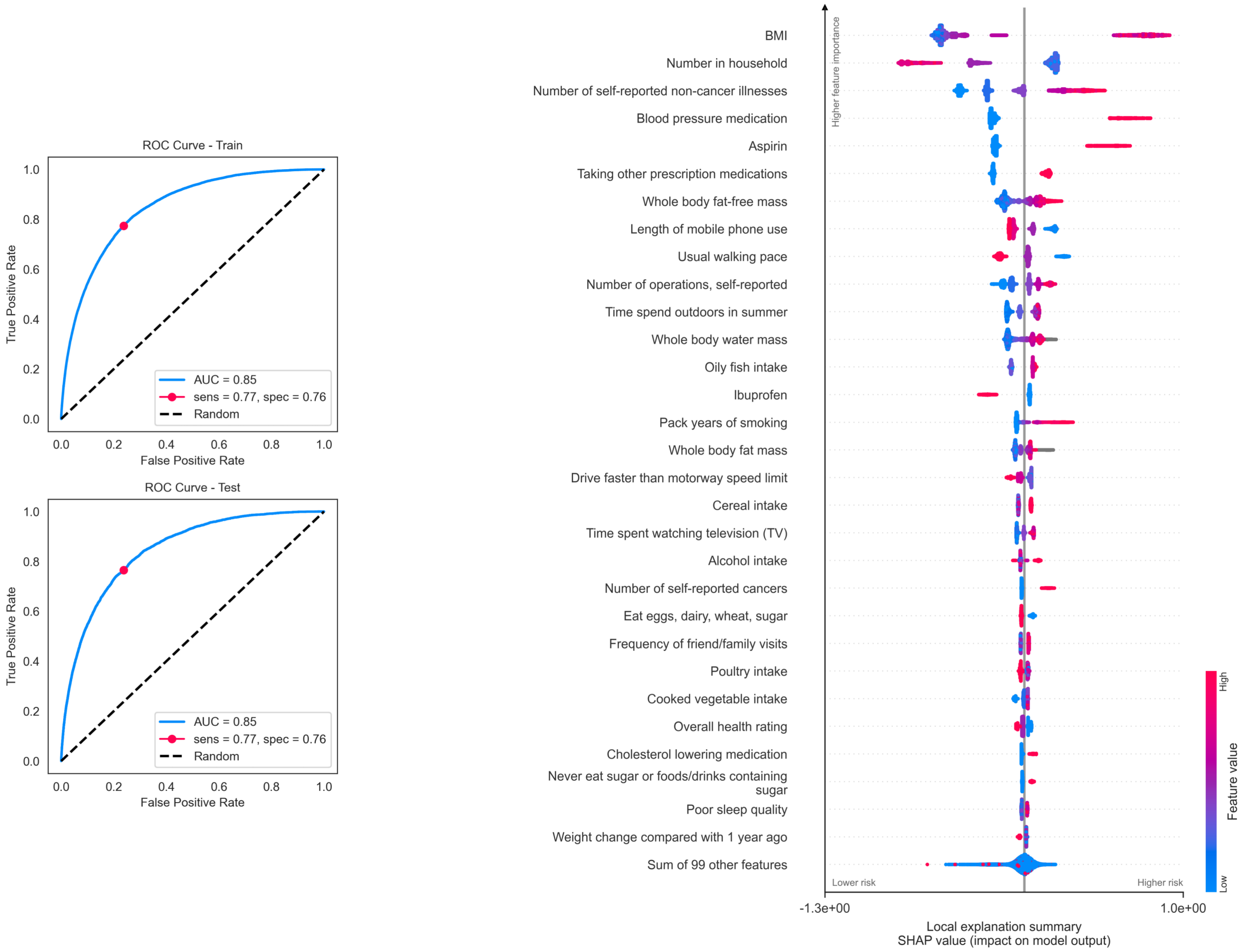

Supplementary Figure 3: Performance and SHAP analysis of classifier trained on modifiable features

Evaluation of the CatBoost classifier trained only on modifiable features. The left panel displays the ROC curves for the training cohort ("Train") and the validation cohort ("Test"). The right panel displays the top 30 modifiable features with the highest mean SHAP values.

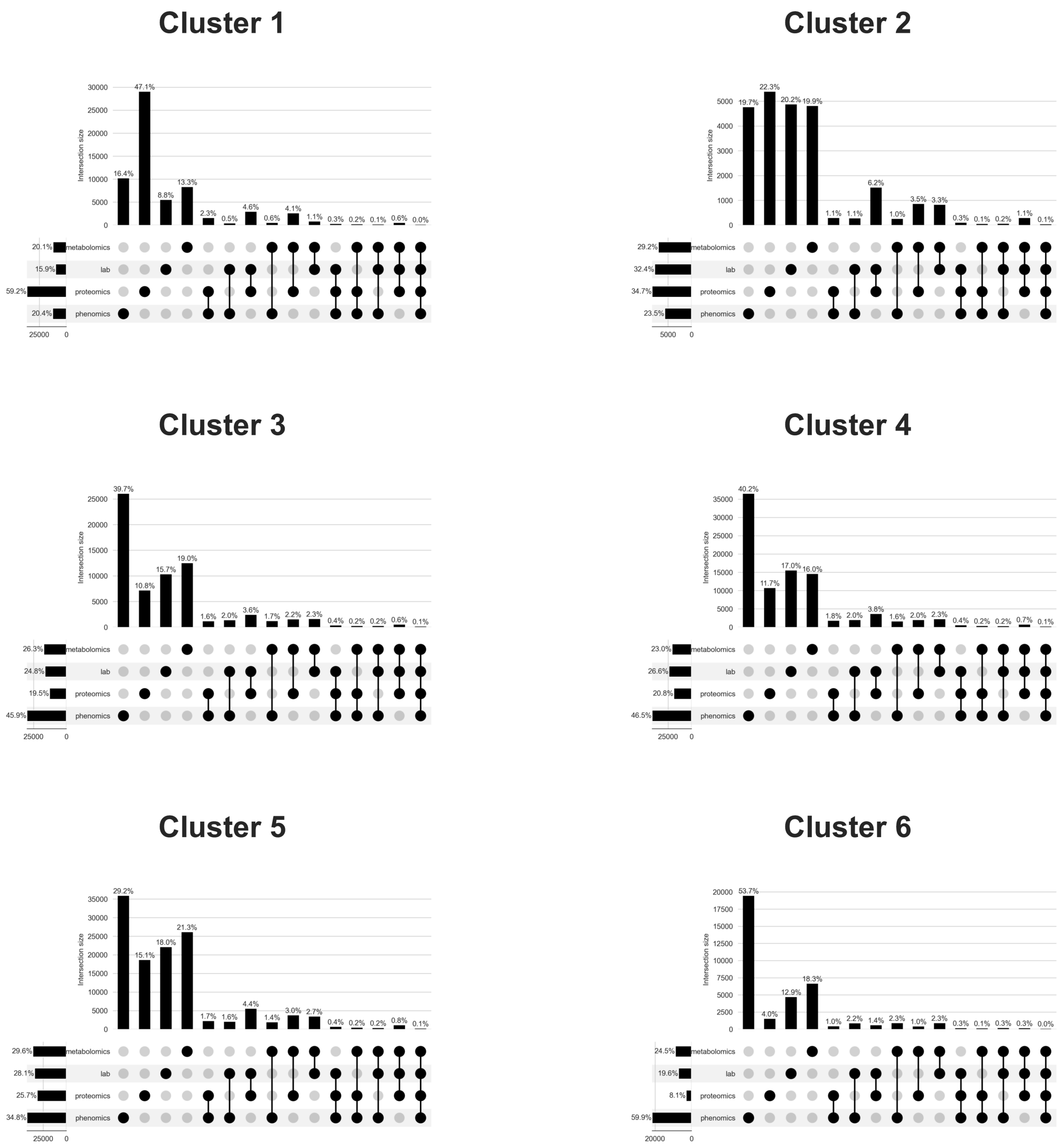

Supplementary Figure 4: Upset plots illustrating similarity overlap among affinity matrices prior to SNF

Upset plots showing the percentage of cases within each HFpEF Cluster that display the highest similarity across all four affinity matrices—proteomics, metabolomics, phenomics, and laboratory measurements—before integration using SNF. For each cluster, the plots depict the intersection of cases with top similarity scores in different data modalities, highlighting the degree of overlap prior to fusion. The percentage of intersected modalities (connected dots) summarises cases that exhibited similarity values within a 10% range across more than one modality.

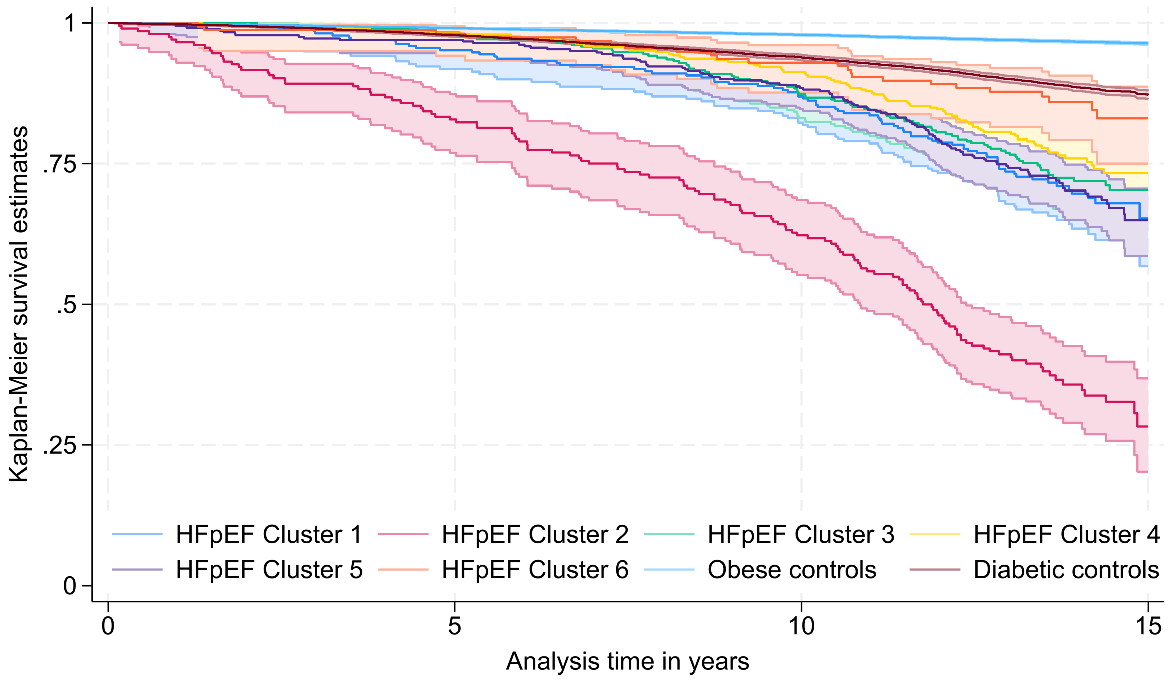

Supplementary Figure 5: Kaplan–Meier survival estimates comparing HFpEF clusters and control groups

Kaplan-Meier survival curves comparing HFpEF clusters, obese controls, and diabetic controls. Note that the Kaplan-Meier plot emphasises early mortality by incorporating the timing of events and censoring. This can result in a steeper decline in the survival curve for clusters with early events, leading to an appearance of higher median mortality compared to the absolute mortality percentage, which does not account for event timing.

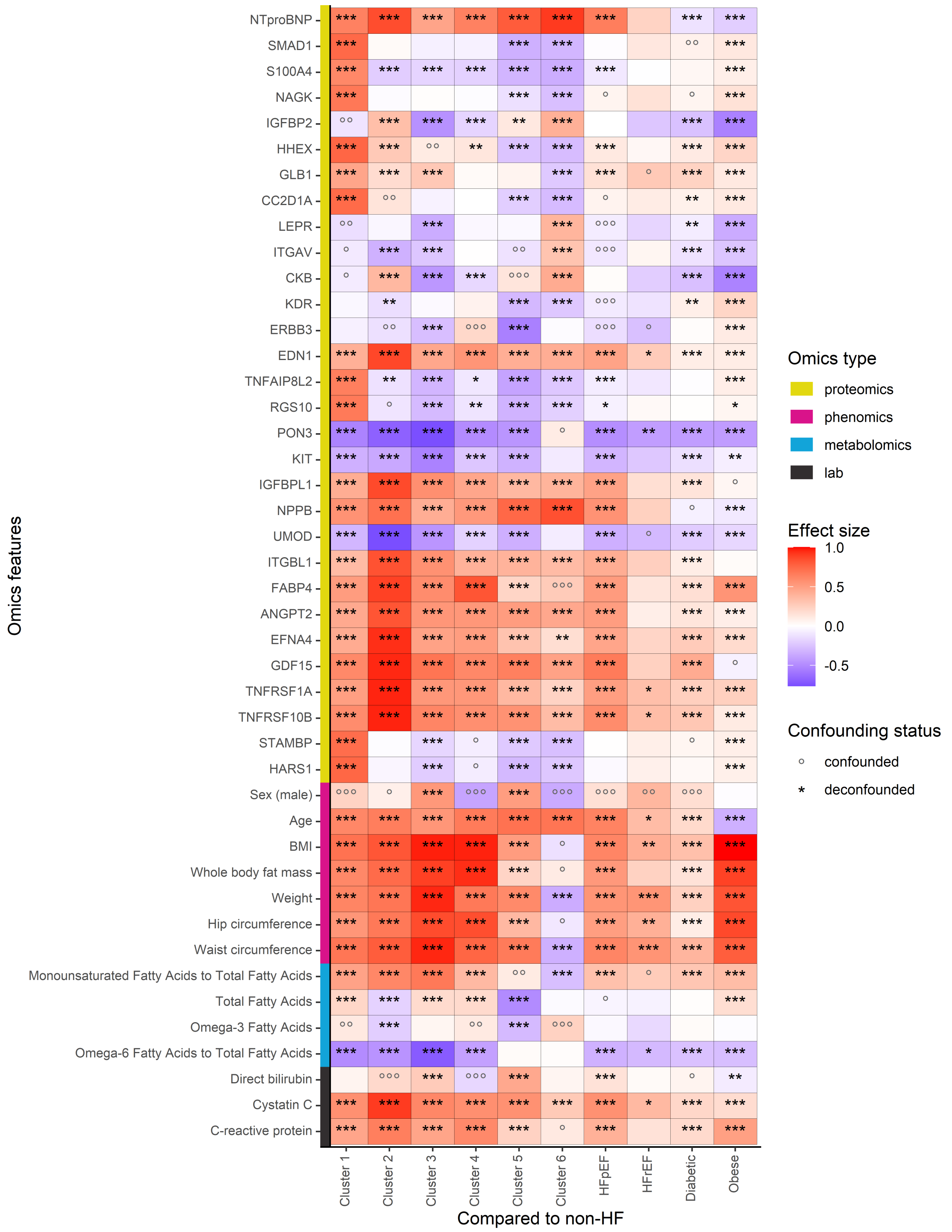

Supplementary Figure 6: Confounding and deconfounding of multi-omics associations with clusters compared to controls

This heatmap displays the omics features that were consistently ranked among the top 50 by mean SHAP value for the cluster-control classifier (see Table S9). For full details on heatmap construction, refer to the caption of Figure 3.

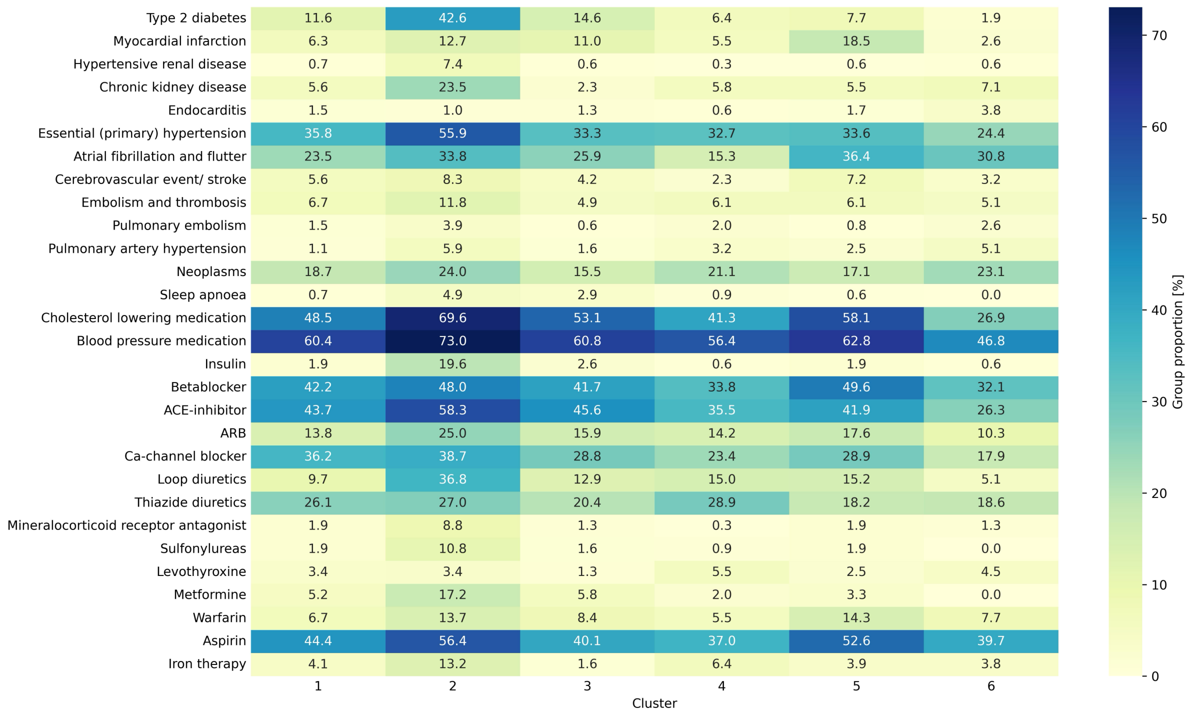

Supplementary Figure 7: Comorbidities and medication use across HFpEF clusters

This heatmap illustrates, for each of the six HFpEF clusters (x-axis), the percentage of patients (colour scale, right) diagnosed with specific comorbidities or receiving particular medication (y-axis).
